# Physiological symmetry of transcranial magnetic stimulation‐evoked EEG spectral features

**DOI:** 10.1002/hbm.26022

**Published:** 2022-07-21

**Authors:** Sasha D'Ambrosio, Diego Jiménez‐Jiménez, Katri Silvennoinen, Sara Zagaglia, Marco Perulli, Josephine Poole, Renzo Comolatti, Matteo Fecchio, Sanjay M. Sisodiya, Simona Balestrini

**Affiliations:** ^1^ Department of Clinical and Experimental Epilepsy UCL Queen Square Institute of Neurology London UK; ^2^ The Chalfont Centre for Epilepsy Chalfont St. Peter UK; ^3^ Neuro Center Kuopio University Hospital Kuopio Finland; ^4^ Department of Neuroscience Catholic University of the Sacred Heart Rome Italy; ^5^ Dipartimento di Scienze Biomediche e Cliniche “L. Sacco” Università degli Studi di Milano Milan Italy; ^6^ Center for Neurotechnology and Neurorecovery, Department of Neurology Massachusetts General Hospital Boston Massachusetts USA; ^7^ Neuroscience Department Meyer Children's Hospital‐University of Florence Florence Italy

**Keywords:** brain stimulation, cortical excitability, neurophysiology, symmetry, TMS‐EEG

## Abstract

Transcranial magnetic stimulation (TMS)‐evoked EEG potentials (TEPs) have been used to study the excitability of different cortical areas (CAs) in humans. Characterising the interhemispheric symmetry of TMS‐EEG may provide further understanding of structure–function association in physiological and pathological conditions. We hypothesise that, in keeping with the underlying cytoarchitectonics, TEPs in contralateral homologous CAs share similar, symmetric spectral features, whilst ipsilateral TEPs from different CAs diverge in their waveshape and frequency content. We performed single‐pulse (<1 Hz) navigated monophasic TMS, combined with high‐density EEG with active electrodes, in 10 healthy participants. We targeted two bilateral CAs: premotor and motor. We compared frequency power bands, computed Pearson correlation coefficient (*R*) and Correlated Component Analysis (CorrCA) to detect divergences, as well as common components across TEPs. The main frequency of TEPs was faster in premotor than in motor CAs (*p* < .05) across all participants. Frequencies were not different between contralateral homologous CAs, whilst, despite closer proximity, there was a significant difference between ipsilateral premotor and motor CAs (*p* > .5), with frequency decreasing from anterior to posterior CAs. Correlation was high between contralateral homologous CAs and low between ipsilateral CAs. When applying CorrCA, specific components were shared by contralateral homologous TEPs. We show physiological symmetry of TEP spectral features between contralateral homologous CAs, whilst ipsilateral premotor and motor TEPs differ despite lower geometrical distance. Our findings support the role of TEPs as biomarker of local cortical properties and provide a first reference dataset for TMS‐EEG studies in asymmetric brain disorders.

## Graphical abstract

TMS‐evoked potentials from homologous cortical areas share similar spectral features, whilst TMS‐evoked potentials from ipsilateral cortical areas have different spectral features. TMS can be used as a potential biomarker to assess cortical functions.

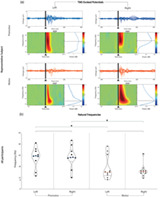

## 1 INTRODUCTION

Transcranial magnetic stimulation (TMS) coupled with EEG (TMS‐EEG) is a controlled perturbational approach that directly activates a target cortical area (CA) to assess its excitability (Ilmoniemi et al., 1997; Rosanova et al., 2009). TMS‐evoked EEG potentials (TEPs) are complex waveforms generated by averaging segments of EEG recording (“trials”) which are time‐locked to the TMS pulses. Typically, TEPs have a duration of hundreds of milliseconds and are characterised by sustained increases of power in specific frequency bands that depend on the CA targeted (Rosanova et al., 2009). When not confounded by scalp muscle artefacts, auditory, or extraneous sensory activations, TEPs provide a reliable read‐out of the reactivity of cortical circuits (Hallett, 2007; Komssi & Kähkönen, 2006; Rogasch & Fitzgerald, 2013; Rosanova et al., 2012).

TMS can be delivered with different pulse configurations, whereby current is either delivered with a bidirectional flow (biphasic stimulation), or dampened after the first quarter cycle, thus delivering a unidirectional current flow (monophasic stimulation) (Sakai et al., 1997).

In TMS‐EEG experiments, either passive or active EEG systems configurations may be used (Mancuso et al., 2021). Active configurations are more sensitive to artefacts due to transient voltage changes; however, signal pre‐amplification directly at the electrode level may allow for better signal quality at higher interelectrode impedance compared to passive systems, and enables faster preparation in the clinical environment (Laszlo et al., 2014).

Previous studies employing biphasic TMS and passive electrodes have shown that TEPs follow a rostro‐caudal gradient in their main oscillatory frequency (i.e., natural frequencies) (Rosanova et al., 2009). This intrinsic frequency gradient at which CAs oscillate is thought to reflect the cytoarchitectonics, as well as the connectivity, of distinct thalamocortical modules (Ferrarelli et al., 2012; Rosanova et al., 2009) and can be altered in brain pathological conditions (Ferrarelli et al., 2012). Up‐to‐date, this gradient has only been described using biphasic TMS and passive electrodes. Furthermore, the interhemispheric symmetry of the natural frequency gradient has never been assessed.

In this work, we sought to determine the symmetry of TEP spectral features by examining the presence of comparable bilateral natural frequencies specific to each CA, to corroborate previous work investigating the similarity of TEPs recorded from homologous regions (Casula et al., 2020; Vallesi et al., 2021). We also assessed the use of monophasic TMS coupled with active high‐density EEG (hd‐EEG) to reproduce the natural frequencies previously determined in premotor areas, and for the first time measured the natural frequencies for the hand region of the primary motor cortex on the left and right hemispheres. Additionally, we assessed the interhemispheric dynamics by applying the interhemispheric signal propagation (ISP) and balance (IHB) as described elsewhere (Casula et al., 2020). We extracted the spectral features of TEPs recorded from 10 healthy individuals in whom we targeted both premotor and motor CAs, and compared TEP natural frequencies and voltages by applying time‐frequency analysis and Pearson's correlation coefficient (*R*), respectively. We also identified the TEP components that were reliably reproducible across participants and specific to the targeted CA by applying the correlated component analysis (CorrCA). Our ultimate goal was to demonstrate that TMS may serve as a potential biomarker to assess cortical activity providing functional information on the underlying cytoarchitectonics, beyond the information provided by standard neuroimaging.

## 2 METHODS

### 2.1 Participants

The study was approved by the local ethics committee (REC ref 15/LO/1642). All participants gave written informed consent. Exclusion criteria were as follows: history of traumatic brain injury, neurological or psychiatric diseases, presence of intracranial metallic implants, drug release dispensers, metallic tattoos or cardiac pacemakers (Rossi et al., 2009). Ten right‐handed healthy participants were recruited to participate in the study (six females; age: median 33.00 years, interquartile range [IQR] 5.00, all with higher education level, Table 1).

**TABLE 1:** Demographic data and information on experimental sessions for all the study participants

Participant ID	Gender	Age at the day of experiment (years)	Handedness	Individual MRI availability	MRI voxel size
S1	Female	35	Right	Yes	160 × 240 ×256
S2	Female	31	Right	Yes	256 × 212 × 256
S3	Male	29	Right	Yes	256 × 256 × 96
S4	Female	33	Right	No	193 × 229 × 193
S5	Female	28	Right	No	193 × 229 × 193
S6	Female	33	Right	Yes	300 × 384 × 384
S7	Male	62	Right	No	193 × 229 × 193
S8	Male	58	Right	Yes	256 × 170 × 256
S9	Male	41	Right	No	193 × 229 × 193
S10	Female	36	Right	No	193 × 229 × 193

### 2.2 Experimental setup

**FIGURE 1:**
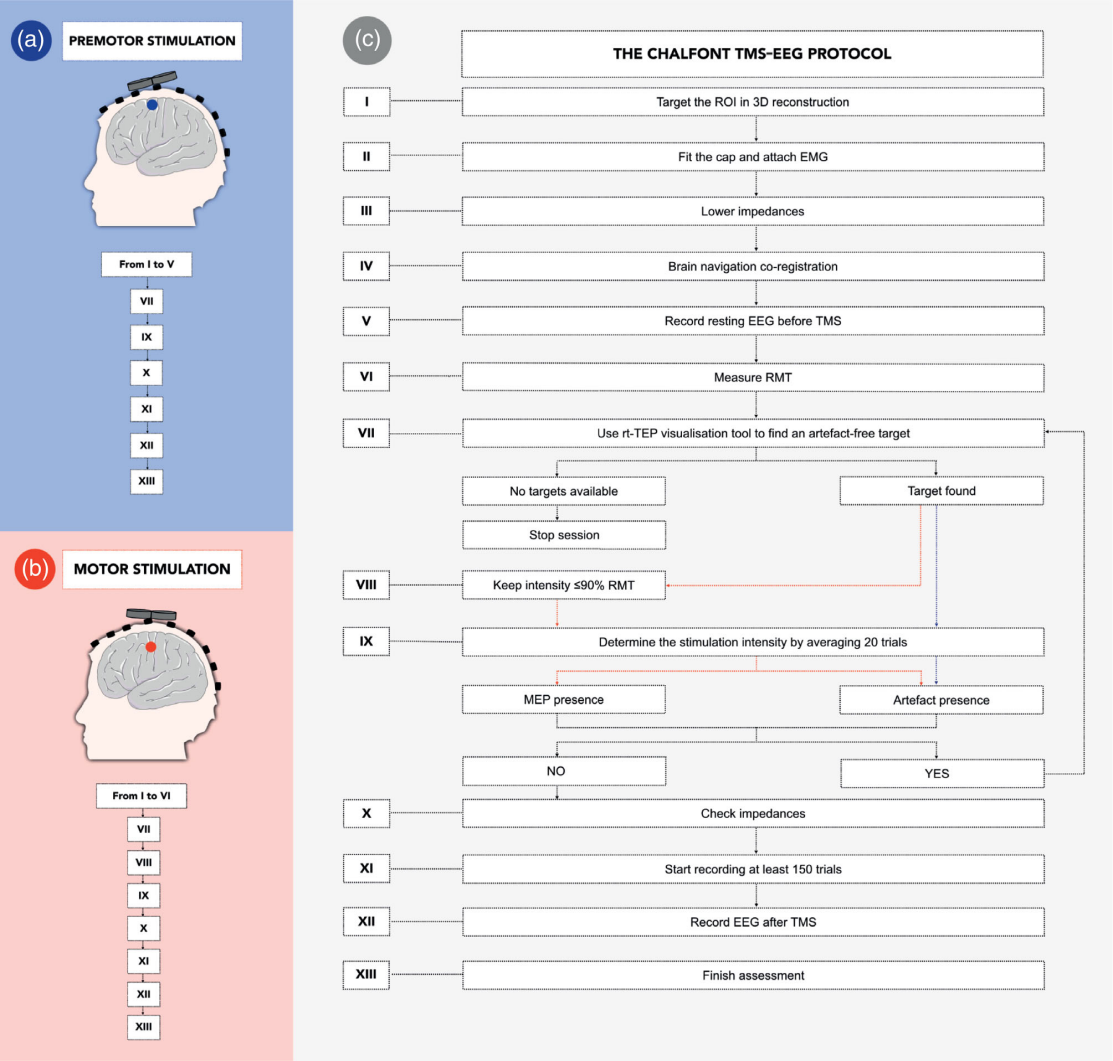
The “Chalfont TMS‐EEG” protocol. Illustration describing our internal protocol for stimulation of premotor (target represented by blue circle, panel a) and motor (red circle, panel b) cortical targets. Panel c illustrates our workflow and can be used as reference to reproduce this work. Additional details of our protocol are provided in the Supplementary Material S1. avg‐ref, average reference; EMG, electromyography; RMT, resting motor threshold; ROI, region of interest; rt‐TEP = real‐time visualisation tool

Experiments were conducted at the Chalfont Centre for Epilepsy, Buckinghamshire, UK, under the research governance of University College London (UCL). We devised a systematic approach for our TMS‐EEG assessment (Figure 1, full details of the protocol are available in the Supplementary Material S1).

### 2.3 Transcranial magnetic stimulation‐click sound masking

To avoid the contamination of TEPs by auditory responses to the click produced by the TMS coil, we adopted the TMS‐click sound masking toolbox *TAAC* (Russo et al., 2022) and participants wore noise‐cancelling in‐ear headphones (Shure SE215‐CL‐E Sound Isolating, for the detailed methodology see Supplementary Material S2).

### 2.4 High‐density EEG recording

For all the experimental sessions, we used hd‐EEG recording following international standards (Nuwer et al., 1998; Sinha et al., 2016). An ActiCHamp 63‐channel amplifier was used, and TMS‐compatible actiCAP active electrodes (Brain Products GmbH, Germany) were placed in the international 10–20 montage referenced to the forehead. The impedance of all electrodes was kept below 5 kΩ (Ilmoniemi & Kičić, 2010). EEG signals were band‐pass filtered between 0.1 and 500 Hz and sampled at 5 kHz with 32‐bit resolution. With this specific equipment, the magnetic artefact induced by the stimulation pulse does not last longer than 5 ms in the EEG recording.

### 2.5 EMG recording and RMT measurement

At the start of each experiment, the motor “hotspot” for the first dorsal interosseus (FDI) muscle was located over the hemisphere contralateral to the muscle being monitored and the resting motor threshold (RMT) was determined for both hemispheres, following Rossini and colleagues' guidelines (Rossini et al., 2015). RMT was considered as the TMS intensity required to elicit motor evoked potentials (MEPs) >50 μV in 5 of 10 trials delivering pulses at 0.2 Hz minimum, measured from the FDI muscle (Supplementary Material S3).

### 2.6 Brain navigation, TMS targeting and brain stimulation

In this work, CA refers to any of the motor or premotor brain regions, whilst we define the region of interest (ROI) as the group of four channels related to each CA under study (F1‐Fz‐Fc1‐Fcz for left premotor, F2‐Fz‐Fc2‐Fcz for right premotor, C5‐C3‐Cp5‐Cp3 for left motor, C4‐C6‐Cp4‐Cp6 for right motor, Table S1). Two bilateral scalp targets were identified within the areas indicated by the four above‐mentioned ROIs. Individual structural magnetic resonance images (MRI, T1‐3D sequence) were available for five participants (Table 1). During the assessments on these subjects, we verified that the two anterior targets were overlapping the caudal frontal superior gyrus (i.e., the premotor—BA6) and that the two posterior targets were overlapping the precentral gyrus (i.e., the motor—BA4) areas. This selection of CAs was based on previous studies that assessed cortical excitability *in vivo* in humans (Ferrarelli et al., 2012). In these subjects, the TMS coil was oriented: (i) overlaying the anatomical targeted MRI area, and (ii) delivering the TMS‐induced current perpendicular to the stimulated cortical gyrus. In order to ensure inter trial reproducibility also for those subjects in which individual MRI was not available, a template MRI from Brainsight software (Rogue Research) was adopted. Finally, we used a Brainsight 3D infrared Tracking Position Sensor Unit to co‐register the TMS coil and the participant's head surface anatomy within the reference space of the individual structural MRI, or of the template MRI.

Stimulation intensity was expressed as a percentage of the maximal stimulator output (MSO), and ranged between 30% and 90% MSO, depending on individual RMT and on the targeted CA. Specifically, the median stimulation intensities used in each CA were: left premotor (71.50%, IQR 10.50), right premotor (76.00%, IQR 15.25), left motor (61.00%, IQR 12.75), right motor (57.00%, IQR 13.75; see Table S1 for individual intensities).

A real‐time visualisation tool (rt‐TEP) was used to guide coil orientation and to determine the stimulator intensity, minimising muscle artefacts and ensuring the presence of a detectable TEP (Casarotto et al., 2022). Stimulation intensity was determined differently depending on the CA under study. For TEPs evoked in the motor CA, intensity was the highest available below ≤90% of the RMT, to avoid possible sensory‐feedback contamination, as previously described (Fecchio et al., 2017). For premotor TEPs, we used the stimulator intensity able to elicit at least a 10 μV‐amplitude response in the average of the first 20 trials. This response was measured in the channels closest to the stimulation site (as per Casarotto et al., 2022), on the first peak‐to‐peak component between 10 and 50 ms after the TMS pulse, using an average reference montage. In this work, we also measured the first TEP component after pre‐processing and averaging. We consistently found intra‐session asymmetric amplitudes with the highest voltages in the channels under the coil, which we considered as a proof of direct cortical perturbation (Belardinelli et al., 2019; Casarotto et al., 2022). Wilcoxon matched pairs signed rank test was used to compare the amplitude of this component between ipsilateral and contralateral homologous CAs across all participants. Values of *p* < .05 were deemed as significant.

A figure of eight coil with 70 mm diameter driven by a monophasic stimulator (Magstim 200^2^) was used for all the experiments. Single pulses were delivered with a randomly jittered median interstimulus interval of 3.57 s (IQR = 0.57). TMS pulses were delivered to the same location with an error of less than 2 mm in each direction and within 2° of the angle of the target stimulus, as guided by the neuronavigation system. During a single TMS‐EEG session, we collected at least 150 trials.

### 2.7 Data pre‐processing

TMS‐EEG data pre‐processing was performed using Matlab R2016a (The MathWorks). First, the TMS artefact was removed from all the trials replacing the recording between −2 and 5 ms from the TMS pulse with the 7 ms of time interval before (between −9 and −2 ms from the pulses). All trials were segmented ±800 ms around the stimulus and high‐pass filtered at 1 Hz (Makeig et al., 2002). Channels containing line‐noise >50 μV lasting more than the 10% of the entire session duration were manually excluded. Subsequently, segments containing more than 50 μV of electrical activity were detected and rejected by visual inspection by two trained researchers (SDA, DJJ). Channels were re‐referenced to the average reference and rejected channels were interpolated using the EEGLAB spherical interpolation function. We used independent component analysis (ICA) to remove any residual artefacts caused by eye movements, scalp muscle activation or electrical interference of devices (EEGLAB *runica* function; Makeig et al., 2002). Finally, data were down‐sampled at 1 kHz, low‐pass filtered (45 Hz, notch 50 Hz) and segmented again in a time window of ±600 ms around TMS pulses (Fecchio et al., 2017; Makeig, 1993). After pre‐processing, we included 10 participants with bilateral TEPs obtained from premotor and motor stimulations for further analysis (Table 1).

### 2.8 Natural frequencies

We considered the natural frequency as the main (i.e., the most powerful) frequency of TMS‐evoked oscillation globally across all channels, between 20 and 200 ms after the TMS pulse (Rosanova et al., 2009). We assessed TEP spectral features by analysing the event‐related spectral perturbation (ERSP). ERSP measures the average dynamic change in amplitude across all the bands of the EEG frequency spectrum in relation to a specific event (Makeig, 1993). We performed time‐frequency decomposition analysis with wavelet transformation (Morlet, 3.5 cycles) between 8 and 45 Hz by computing the EEGLAB *Newtimef* function (Grandchamp & Delorme, 2011), within the Matlab‐based public license toolbox EEGLAB (Delorme & Makeig, 2004). Absolute spectral normalisation was conducted on all trials by performing a full‐epoch length correction. A pre‐stimulus baseline correction between −500 and −100 ms from the pulse was applied to the pre‐processed data, with the time window set 100 ms apart from the stimulus in order to avoid any possible post‐stimulus contamination. The *Newtimef* function computes the surrogate distribution (i.e., creates timepoints with values randomly chosen from the baseline) at each frequency by permuting real baseline values. Thus, it tests whether the original ERSP value points are present in the 99.5% tail of the surrogate distribution of any given frequency (Fecchio et al., 2017), in which case each specific time–frequency point is considered significant at α < .01 (Fecchio et al., 2017), after correction for multiple comparisons, and using the false discovery rate (FDR) procedure (Grandchamp & Delorme, 2011). We calculated the average of the most powerful evoked frequency for each TMS session performed in the four CAs under study, as in Rosanova et al. (2009). We applied the Wilcoxon matched pairs signed rank test for comparing the natural frequencies between ipsilateral and contralateral homologous TEPs across all participants. Values of *p* < .05 were deemed as significant. We calculated natural frequencies evoked at the local level by narrowing down the number of averaged channels to the ROI (four channel selection) band to the one channel closest to the coil (see Table S1, and Supplementary Material S4). We also compared the distribution of natural frequencies between subjects where their individual MRI was used for neuronavigation versus subjects where the MRI template was used. We used the Wilcoxon signed rank test for comparison of all subjects, and then divided the cohort according to the CA stimulated. Values of *p* < .05 were deemed to be statistically significant.

### 2.9 Correlation coefficient analysis

We measured the degree of correlation between the voltages of the TEPs, comparing both ipsilateral and contralateral homologous intra‐participant conditions. Specifically, we calculated the correlation coefficient (*R*) by using the function *corrcoef* from Matlab R2016a (The MathWorks). For this purpose, after averaging the four channels of each selected ROI, *corrcoef* was applied to compare the waveform distribution obtained from the ROI average in the time range between 20 and 200 ms after the stimulus (the same time range used in previous analysis). Therefore, we measured four *R*s in each participant, comparing voltages from: (i) left premotor and right premotor TEPs; (ii) left motor and right motor TEPs; (iii) left premotor and left motor TEPs; (iv) right premotor and right motor TEPs. Moreover, we tested the *R* distribution across participants, by applying the Kruskal–Wallis test across the *R*s obtained. Values of *p* < .05 were deemed as significant. Finally, we calculated which *R* distributions statistically differed from one another by applying the Dunn's statistics across *R*s.

Since we adopted two different approaches for tuning the stimulation intensity across different areas, we controlled for the possible association between the natural frequencies and the stimulation intensities used. To this aim, we calculated the correlation coefficient between the absolute values of natural frequencies and intensities used in each area under stimulation. We then conducted a linear regression analysis of the distribution (*y* = α + β*x*, where *y* is intensity and *x* is the natural frequency) to further analyse the association between intensity stimulation and natural frequencies. Values of *p* < .05 were deemed as significant.

### 2.10 Correlated component analysis

We applied CorrCA (see Supplementary Material S5 for details) to understand whether reproducible components were shared over homologous TEPs. Hence, we took N repetitions as participants, hd‐EEG channels as dimensions D, and T as time samples. We then computed CorrCA between 20 and 200 ms from the TMS pulse in the four groups of TEPs obtained from each CA. The Python code for computing CorrCA is available at https://github.com/renzocom/CorrCA and is based on the original Matlab implementation by Parra et al. (2018). Statistical significance was assessed using random circular shuffle (400 surrogates, alpha = 0.05). Moreover, we wanted to assess the presence of bilateral reproducible components in premotor and motor areas. To this aim, we combined TEPs from both hemispheres by flipping the channel location of the right premotor and right motor TEPs to the left. Therefore, we computed CorrCA between 20 and 200 ms from the TMS pulse on “20 left premotor” (10 left premotor + 10 right premotor flipped to the left) and “20 left motor” (10 left motor + 10 right motor flipped to the left) TEPs.

### 2.11 Interhemispheric signal propagation and inter hemispheric balance

To assess the interhemispheric transmission, we first analysed the TMS‐evoked response on the stimulated hemisphere and on the contralateral. Specifically, we measured the signal propagation of the TEP activity from the stimulated hemisphere to the nonstimulated hemisphere by applying ISP and IHB analysis of motor TEPs. To do this, we replicated the methodology reported by Casula et al. (2020). The ROIs for this calculation were selected based on previous TMS‐EEG studies (Casula et al., 2020; Määttä et al., 2017). We applied the Wilcoxon matched‐pairs signed‐rank test to compare the ISP between left and right motor cortices across all participants. Values of *p* < .05 were deemed as significant.

## 3 RESULTS

We performed single‐pulse navigated monophasic TMS coupled with active hd‐EEG in 10 healthy participants. Each subject underwent to four TMS‐EEG sessions. A total of 40 sessions were included in the following results.

### 3.1 Natural frequencies

**FIGURE 2:**
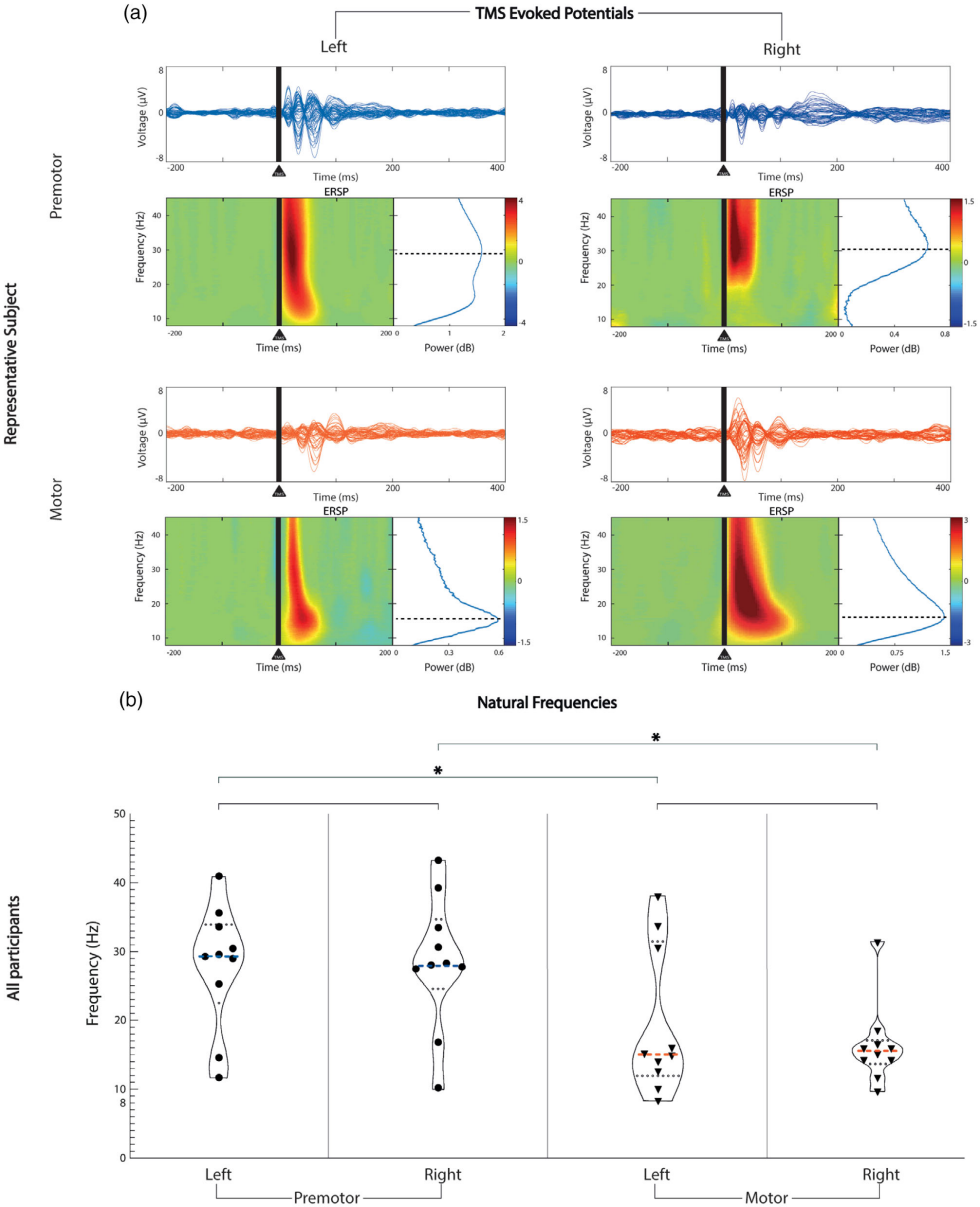
Natural frequency comparison between contralateral homologous and ipsilateral CAs. Horizontal lines represent statistical comparisons. (a) For a representative participant (P1), at the top of each area, we show the channel voltages averaged across trials between −200 and 400 ms from the stimulus, on a scale between −8 and 8 μV for each area (butterfly plots). Under each butterfly plot on the left, we present the time‐frequency decomposition averaged across all the channels between 8 and 45 Hz. At the bottom right of each butterfly plot, we show the cumulative ERSP from 20 to 200 ms. (b) Violin plot showing the full distribution of natural frequency data for each area derived from panel a across all participants. The median is represented by the bold dashed line in each plot. The median resulted from left premotor is 29.37 Hz, from right premotor is 27.94 Hz, from left motor is 15.03 Hz, from right motor is 15.61 Hz. Dotted lines represent the quartiles distribution of the natural frequencies for each area. Blue: premotor CAs, red: motor CAs; _*_, statistically significant comparisons; CA, cortical area; ESRP, event‐related spectral perturbation

We found ipsilateral differences in the natural frequencies, which were higher in the premotor (left and right median 29.09 Hz, IQR 7.74 Hz) than in motor CAs (left and right median 15.18 Hz, IQR 5.16 Hz). Across participants, the TEP median natural frequency was 29.37 Hz (IQR 11.47 Hz) in the left premotor cortex and 27.94 Hz (IQR 10.11 Hz) in the right premotor cortex. No significant interhemispheric differences were found between the natural frequencies of premotor (sum of signed ranks [*W*] = 10, *p* = .6426) or motor CAs (left motor median 15.03 Hz, IQR 14.05 Hz vs. right motor median 15.61 Hz, IQR 2.15 Hz, *W* = −6.00, *p* = .7871). However, significant differences were found between ipsilateral premotor and motor CAs both on the left (median 29.37 Hz vs. 15.03 Hz, *W* = −45.00, *p* = .0195), and on the right (median 27.94 Hz vs. 15.61 Hz, *W* = −39.00, *p* = .0488, Figure 2). We obtained similar results when comparing natural frequencies calculated at the local level (full results available in the Supplementary Material S6, Figure S1, Table S2). Additionally, we found that variability in the natural frequencies was not affected by the use of individual MRI versus template (details in Table S3).

### 3.2 Correlation coefficient analysis

**FIGURE 3:**
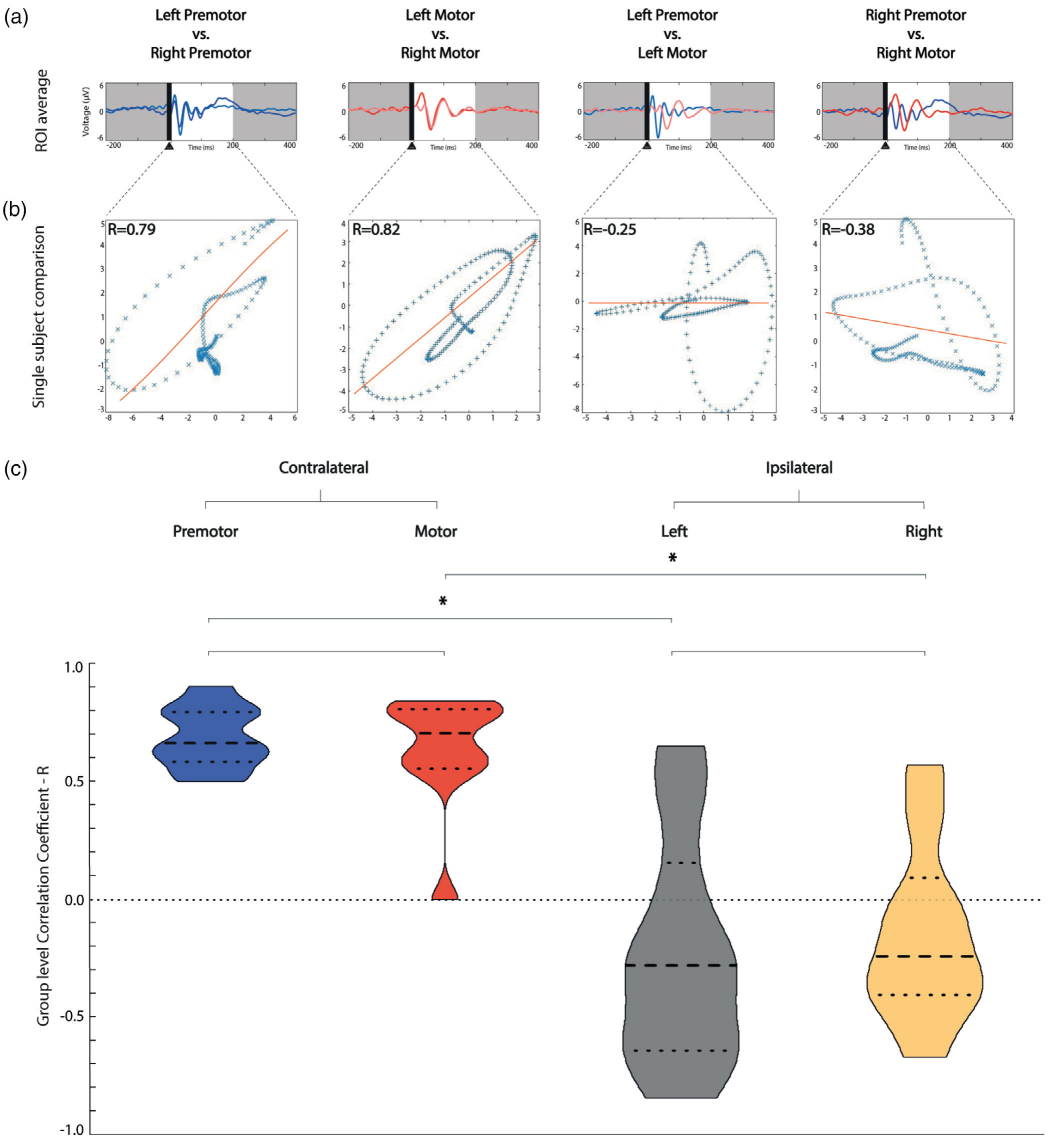
Correlation coefficient is higher when comparing contralateral homologous TEPs than ipsilateral TEPs. (a) Comparison of voltage distribution over time on the average of the four channels closest to the stimulation (ROI) for one representative participant (P1). We represented left premotor stimulation in light blue, right premotor stimulation in dark blue, left motor stimulation in light red, right motor stimulation in dark red. (b) This panel shows the association analysis of the voltages of the evoked potentials presented in panel a between 20 and 200 ms from the TMS pulse, using the Pearson's correlation coefficient. (c) Violin plot with median and interquartile range (bold and light dashed lines, respectively) of the intra‐participant correlation coefficient calculations. Contralateral homologous CAs correlation coefficients are represented in the two violin plots on the left, ipsilateral correlation coefficients are represented in the two violin plots on the right. Horizontal lines represent statistic tests, * represents significant difference. Premotor contralateral homologous comparisons are coloured in blue, motor contralateral in red, ipsilateral left in grey, ipsilateral right in yellow. CA, cortical area; ROI, region of interest; TEP, TMS‐evoked EEG potential; TMS, transcranial magnetic stimulation

The Pearson's coefficient (*R*) analysis showed higher correlation between contralateral homologous ROI comparisons (Figure 3): left premotor versus right premotor, median *R* = .67 (IQR 0.21); left motor versus right motor, median *R* = .71 (IQR 0.25), than between ipsilateral comparisons: left premotor versus left motor, median *R* = −.28 (IQR 0.79); right premotor versus right motor, median *R* = −.24 (IQR 0.49). We found a statistically significant difference between the *R*s median (*p <* .0001, Kruskal–Wallis statistic = 26.26). Dunn's multiple comparison tests of *R*s are shown in Supplementary Material S7.1. We did not find any significant association between the absolute natural frequency values and TMS intensities (detailed results provided in Supplementary Material S7.2).

### 3.3 Correlated component analysis

**FIGURE 4:**
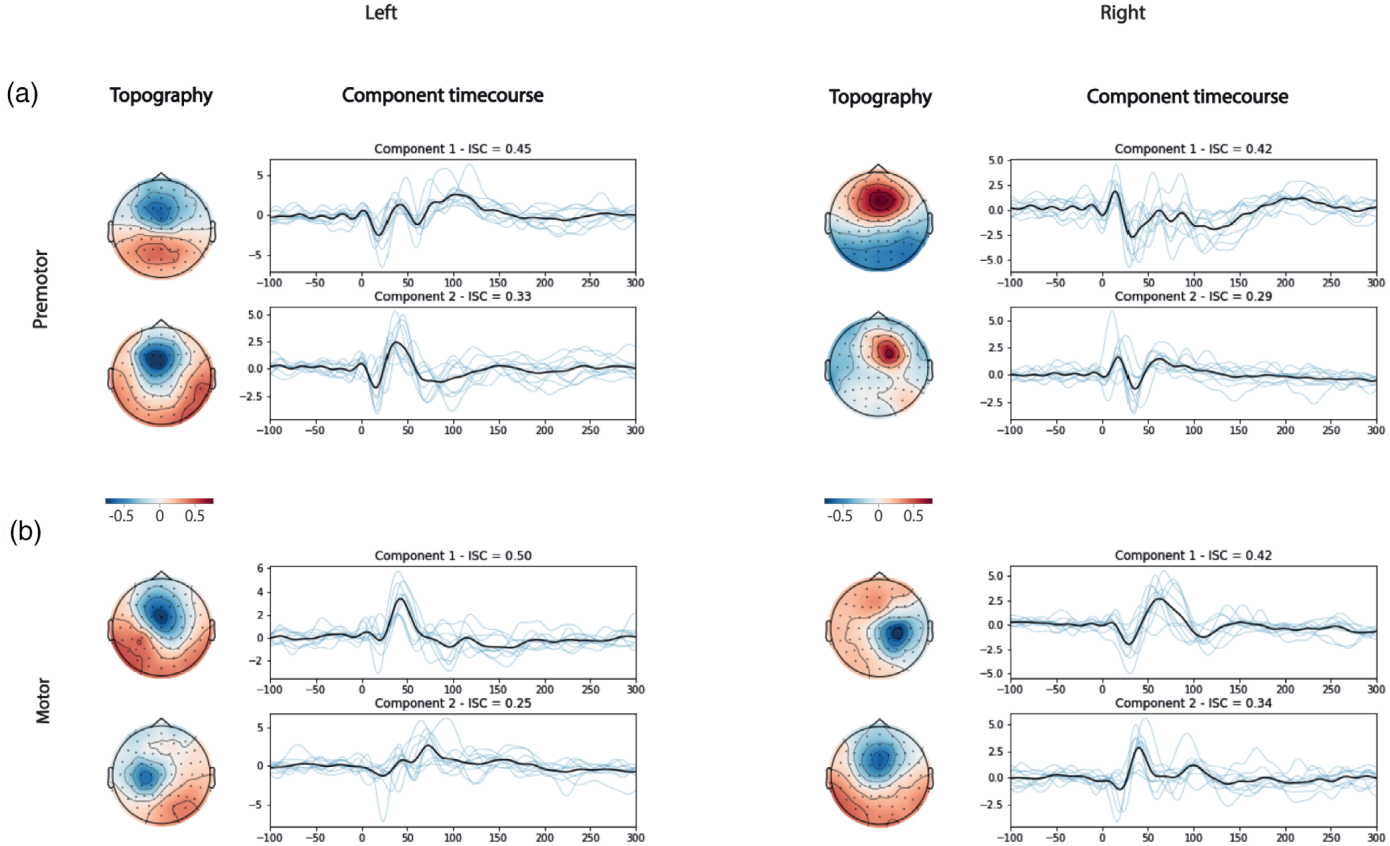
Correlation component analysis for each cortical area. Here, we show the most reproducible components between 20 and 200 ms (participant level components in blue and average in black) and the topographies of their forward model. For each CA, we obtained two reproducible components. The *x* axis represents the time in milliseconds and the *y* axis shows the voltage in microvolts. On the top of each box, we show the ISC for each component. (a) Most reproducible components obtained from TMS‐evoked responses while stimulating the premotor cortex. (b) Most reproducible components obtained from TMS‐evoked responses while stimulating the motor cortex. CA, cortical area; ISC, inter‐participant correlation; TMS, transcranial magnetic stimulation

We calculated the most reproducible components of premotor and motor TEPs in both hemispheres. In line with our hypothesis, they were similar contralaterally and diverged ipsilaterally. Specifically, we found two principal reproducible components in the left and two in the right premotor TEPs. In the left premotor area, the most reproducible component (ISC = 0.45) presented three peaks within the first 100 ms, and was located in the premotor area; the second component (ISC = 0.33) was smaller in power and showed two peaks within the first 100 ms, It was located in the premotor area. In the right premotor area, the most reproducible component (ISC = 0.42) had three peaks within the first 100 ms, and was located in the midline; the second component (ISC = 0.29) was smaller in power and showed two peaks within the first 100 ms, it was located in the right premotor area. Left motor TEPs were characterised by two principal components. Specifically, the most reproducible component (ISC = 0.50) showed one peak within the first 100 ms and was located in the midline, the second component (ISC = 0.25) was smaller in power and presented two peaks within the first 100 ms, it was located in the left motor area. Right motor TEPs were characterised by two principal components. The most reproducible component (ISC = 0.42) showed two peaks, within the first 100 ms, and was located in the right motor area; the second component (ISC = 0.34) was smaller in power and presented two peaks within the first 100 ms, it was located in the midline area (Figure 4).  In Supplementary Material S8 and Figure S2 we present the results of CorrCAs with right and left CAs merged together (i.e.right CAs were flipped to the left).

### 3.4 Amplitude of the first TEP component after pre‐processing and averaging

For this component, we found larger amplitude in premotor TEPs than motor TEPs. The median first component amplitude was 6.91 μV (IQR 6.44) for left premotor TEPs, 7.38 μV (IQR 4.16) for right motor TEPs, 2.67 μV (IQR 4.37) for left motor TEPs, and 2.00 μV (IQR 2.05) for right motor TEPs. When comparing left and right hemisphere TEPs, there was no significant statistical difference for premotor (*W =* −5.00, *p* = .8457) or motor (*W* = −23.00, *p* = .2754) TEPs. There was a significant difference between left ipsilateral premotor and motor TEPs (*W* = −39.00, *p* = .0488) and between right ipsilateral premotor and motor TEPs (*W* = −53.00, *p* = .0039).

### 3.5 Local mean field power

In order to control for the potential confound of the stimulation impact on the reactivity of each stimulated CA, we calculated and compared the local mean field power (LMFP) of each TEP (detailed methodology and results in Supplementary Material S9, Figure S3). We did not find any statistically significant effect (*p* = .7893).

### 3.6 Stimulation distances

**FIGURE 5:**
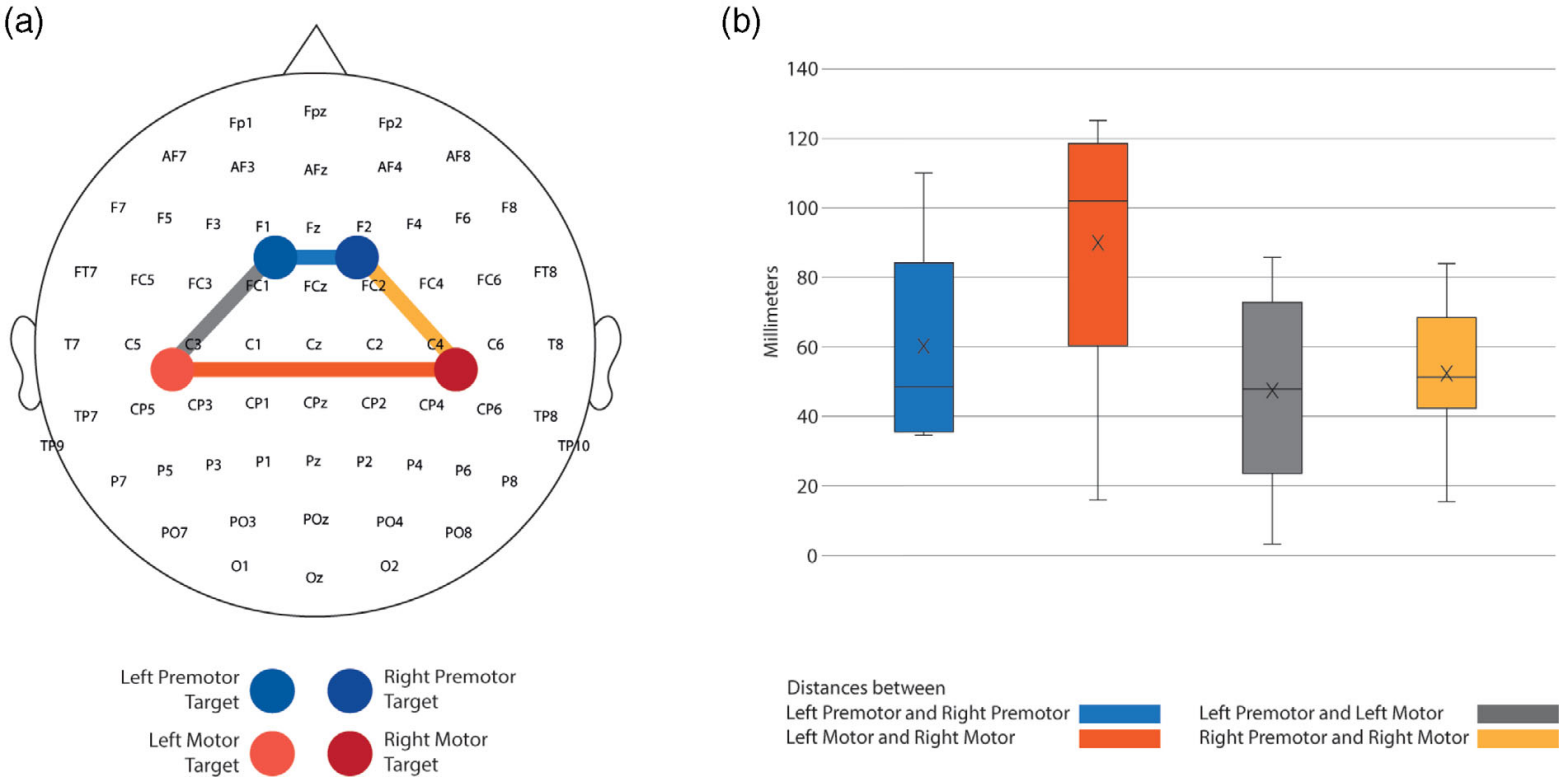
Relative stimulation distances and locations. We used the coordinates from the brain navigation software to calculate the distances between ipsilateral and contralateral homologous target stimulations. (a) Topography of all the targets that we stimulated across participants. The interconnected coloured lines represent the distances we calculated between different targets. Blue represents the distance between left premotor and right premotor targets. Red represents the distance between left motor and right motor targets. The grey colour coding shows the distances between left premotor and left motor, and yellow represents the distance between right premotor and right motor. (b) Box plot of the average of the distances in all the targets we stimulated across all participants. The black line crossing the bars represents the median of each target. The cross inside the bars represents the mean of each target.

We calculated and compared the distances between stimulation sites to control for the location of the stimulations as potential confounder of both gradient and symmetry of TEPs (detailed methodology in Supplementary Material S10). The median distance between premotor areas was 48.45 mm (IQR 46.62 mm), whilst the median distance between motor areas was 101.9 mm (IQR 58.02 mm). The median distance between premotor and motor targets was 47.88 mm (IQR 49.26 mm) on the left, and 51.27 mm (IQR 29.0 mm) on the right (Figure 5). Significant differences were found when comparing contralateral motor versus premotor distances, and controlateral motor versus right or left ipsilateral areas, whereas no statistical differences were revealed by the other comparisons. The results of the comparisons were as follows: (1) distance between motor contralateral homologous areas versus distance between premotor contralateral homologous areas (*W* = 39.00, *p* = .0488); (2) distance between motor contralateral homologous areas versus distance between left ipsilateral areas (*W* = −41.00, *p* = .0371); (3) distance between motor contralateral homologous areas versus distance between right ipsilateral areas (*W* = −49.00, *p* = .0098); (4) distance between left versus right ipsilateral areas (*W* = 23.00, *p* = .2754); (5) distance between premotor contralateral homologous areas versus distance between left ipsilateral areas (*W* = −1.00, *p* = >.9999); (6) distance between premotor contralateral homologous areas versus distance between right ipsilateral areas (*W* = −1.00, *p* = >.9999, Figure 5).

### 3.7 Interhemispheric signal propagation and interhemispheric balance

We calculated the ISP from the left and right motor cortices. We found a consistent interhemispheric inhibition across all our participants (see Supplementary Material S11).

## 4 DISCUSSION

### 4.1 TEP differentiation and symmetry

Here, we used single‐pulse (<1 Hz) navigated monophasic TMS to stimulate bilateral cortical areas and simultaneously record hd‐EEG with active electrodes. Using a different TMS pulse configuration and a different amplifier to those in previous reports, we first reproduced previous findings showing the presence of distinct frequencies evoked across different CAs perturbed by direct transcranial magnetic pulses (Sakai et al., 1997; Sommer et al., 2006). This result confirms the existence of a specific frequency tuning, called the natural frequency, that is intrinsic to the targeted circuit and independent of the specific stimulation and recording set‐up (Ferrarelli et al., 2012; Rosanova et al., 2009). Most importantly, we find that such tuning is symmetric across the two hemispheres.

LMFP analysis showed no significant difference in the response to TMS between 20 and 200 ms across the different stimulation targets. Therefore, the natural frequencies observed are unlikely to reflect differences in the overall effectiveness of the TMS perturbation. Our results showed symmetrical natural frequencies and a high correlation coefficient between contralateral homologous ROIs (Figures 2 and 3). We found significant differences when comparing the distance between homologous contralateral motor areas (which was higher) and the other distances (Figure 5). However, motor TEPs showed symmetrical spectral frequencies despite higher geometrical distance between the contralateral homologous motor targets, whilst ipsilateral motor and premotor TEPs were different despite their closer proximity. These findings confirm that differences in the TEPs features depend more on the cytoarchitectonic differences of the underlying cortex rather than on the geometrical distance between the stimulated areas. Cytoarchitecture and histology are known to be similar in homotopic contralateral areas but can differ substantially at small distances along the anterior–posterior axis (Zilles et al., 2015). In line with this, the fact that TEP spectral features diverged more amongst targets that were geometrically closer on the scalp but more different in terms of underlying cortical cytoarchitecture has important implications for the methodological debate on the origin of TEPs. Indeed, this result can be more parsimoniously explained as the effect of a direct cortical activation, rather than in terms of nonspecific sensory responses to scalp or auditory stimulation. These findings support the role for TMS‐EEG in the study of electrophysiological correlates of the local structural and functional arrangement of cortical circuits. Importantly, our findings of contralateral TEP symmetry only relate to frequency spectra and not amplitude. TEP amplitude amongst contralateral homologous CAs has been previously shown to be rather asymmetric during the same stimulation session, and this has been considered as a proof of direct cortical activation (Belardinelli et al., 2019). We confirmed this after applying ISP analysis (Casula et al., 2020; Hui et al., 2021) to the motor areas (see Supplementary Material S11).

The present study represents the first systematic comparison of the inter‐ and intra‐hemispheric spectral features of TEPs within the same subjects. In 2020, Vallesi and colleagues showed symmetrical TEPs evoked from stimulation of left and right dorsolateral prefrontal cortex (DLPFC) of 12 healthy participants (six females, mean age = 33.0 years, SD = 2.87 years, range = 28–36 years; Vallesi et al., 2021). They described the relative spectral features in terms of ERSP calculated both at the scalp and at the source level and found no significant difference between natural frequencies evoked in the left and right DLPFC. However, the assessment of DLPFC might be confounded by indirect multisensory responses (i.e., auditory and somatosensory) and muscle artefact (Vallesi et al., 2021), a problem that we avoided in anterior CAs by stimulating areas that are far from scalp muscles. Casula and colleagues recently validated the two novel indices of ISP and IHB by using TMS‐EEG, providing the first evidence of correlation between ISP and IHB. In their recent study, they carried out TMS‐EEG in 50 healthy participants, stimulating the motor (M1) cortex bilaterally. The resulting TEPs were found to be symmetrical, as measured by ISP and IHB (Casula et al., 2020) whilst these measures showed to be asymmetric in patients with schizophrenia, as demonstrated by Hui et al. (2021). We replicated similar findings for ISP and IHB calculations applied to motor cortices in healthy subjects (Casula et al, 2020), enhancing the reproducibility value of these two indices. Lastly, most studies report stimulation of M1 at or above the RMT to date. Here, we used stimulation intensities below the RMT for each motor stimulation, in order to avoid eliciting MEPs, which can contaminate the TEP signal via indirect stimulation of the sensory system (Fecchio et al., 2017).

### 4.2 Decomposing TEPs with CorrCA

CorrCA identified TEP components that were reliably reproducible across participants and specific to the stimulated region. CorrCA differentiated temporal and spatial features visible at the voltage level into separate and reproducible components. When merging left and right TEPs together (Supplementary Material S8), the main difference between motor and premotor CAs was a fast (>20 Hz) and reproducible component, present only in premotor TEPs (the fourth premotor component, Supplementary Figure S2). Interestingly, this difference reflects a faster natural frequency in anterior areas with respect to posterior regions and might be attributable to structural differences between the two areas. On the other hand, we found that the first component of the premotor TEPs was superimposable to the first component detected in motor TEPs (ISC = 0.44 in both conditions, Supplementary Figure S2). Although we cannot rule out a contribution of sensory activations due to the perception of the TMS pulses to this common component, it is interesting to note that premotor and motor CAs are part of the same thalamocortical module (Alexander et al., 1991) and sensorimotor network (Melnik et al., 2017). Therefore, the first component shared by premotor and motor TEPs might reflect the activity of the same low‐dimensional neuronal population underpinning the response in both areas. Previous work demonstrated that EEG can detect behavioural features of low‐dimensional neuronal populations of the sensorimotor network (Bisogno et al., 2021; Georgopoulos et al., 1982). Further studies are needed to better understand the putative mechanism responsible for this component.

### 4.3 Monophasic stimulation and hd‐EEG—technical considerations and limitations

We showed that the use of active EEG coupled with a monophasic stimulator can replicate the high‐quality TEPs which have been obtained using the traditional methodology reported in the literature, that is, passive electrodes and biphasic stimulator. The use of active electrodes has the advantage of a faster preparation time and is therefore potentially useful in a clinical setting where the duration of the experiment is a critical factor. The feasibility of TEPs recordings with active electrodes in comparison with passive electrodes has already been demonstrated (Mancuso et al., 2021). In this study, active electrodes facilitated fast preparation and reliable maintenance of impedances below 5 kΩ. However, we observed voltage fluctuations after long stimulation sessions, as previously reported (Laszlo et al., 2014). The use of active electrodes has been associated with more electrical artefact than passive systems, causing a larger decay artefact after the stimulation (Laszlo et al., 2014). We limited the decay artefact by consistently maintaining impedances as low as possible in this study.

Notably, we used online brain navigation instead of standardised coordinates, enabling precise targeting of cortical areas (<2 mm of error). Furthermore, we adopted a real‐time TEP visualisation approach (Casarotto et al., 2022), instead of the typical RMT‐driven technique, allowing for better control of the necessary intensity, recording of genuine TEPs, and avoidance of muscle and electrical artefacts.

Another potential limitation is represented by the systematic adoption of lower intensities for motor stimulations. Saari et al. (2018) previously showed how TEPs change in relation to the intensity used. We conducted a correlation analysis between stimulation intensity and natural frequencies across each stimulated area and we did not find any statistical significance (detailed results in Supplementary Material S79.2). This can be ascribed to three main reasons: (1) the difference between the intensities we used was too little; (2) the range of intensities we used (from ~90% to ~110% RMT) was within a window in which TEP differences are more subtle (in fact the higher differences in TEP shapes in the work by Saari et al. (2018) is found in respect to intensities below 80% of RMT); (3) spectral features are maintained despite changes in the intensities adopted. As such, further work is needed to better understand the impact of TMS intensities on TEP spectral features.

To quantify the features of TEPs in this normative dataset, we included measures of spectral features, correlation coefficient, and correlated component analysis. In future studies, it would be interesting to apply a more fine‐grained analysis of TEP components (peak and latencies) similar to that typically employed in works targeting the motor cortex (Ter Braack et al., 2019).

Finally, MRI data were not available for five participants, so an MRI template was used for the navigation. We checked that the use of individual MRI versus MRI template did not affect the natural frequency variability, although it may have affected the variability of the first component amplitude. Recent findings have suggested that brain responses to TMS can be equivalent when using an MRI template if a sufficient number of registration points are used during the curvilinear reconstruction of the participant's head (Fleischmann et al., 2020).

### 4.4 Future directions

We demonstrate that TEPs are symmetrical in contralateral homologous premotor and motor areas, but asymmetrical in the same areas ipsilaterally, with higher evoked frequencies in the premotor than in motor CAs. Neurophysiological properties measured by TMS‐EEG could reflect the functional architecture of the underlying cortical structures and are possibly determined by the underlying neuronal networks. Our findings support the use of TMS as a potential biomarker to assess cortical function in people with lateralized brain pathologies, such as typically seen in many focal epilepsies or stroke. Our methodology may offer a measure of underlying cortical dysfunction, even in the absence of obvious structural abnormalities, providing an in vivo dynamic assessment of subtle circuit alterations. A normative dataset of TEPs obtained from multiple bilateral cortical targets, with high temporal and spatial resolution, may provide the foundation for future studies investigating focal brain disease and the effect of treatment strategies on a longitudinal basis.

## AUTHOR CONTRIBUTIONS

Sasha D'Ambrosio and Diego Jiménez‐Jiménez: drafting, analysis and data collection; Katri Silvennoinen, Sara Zagaglia and Marco Perulli: data collection; Renzo Comolatti and Matteo Fecchio: data analysis; Simona Balestrini and Sanjay M. Sisodiya: critical review of the manuscript.

## FUNDING INFORMATION

This work was funded by the National Brain Appeal (NBA). Sasha D'Ambrosio, Simona Balestrini and Sanjay M. Sisodiya are supported by the Epilepsy Society. Simona Balestrini is supported by the Muir Maxwell Trust. Part of this work was undertaken at University College London Hospitals, which received a proportion of funding from the NIHR Biomedical Research Centres funding Scheme. Katri Silvennoinen is supported by a Wellcome Trust Strategic Award (WT104033AIA). Renzo Comolatti and Matteo Fecchio are supported by the Tiny Blue Dot Foundation. UCB provided financial support for Sasha D'Ambrosio and Sara Zagaglia. UCB had no editorial control and no input or decision over the selection of authors or topics discussed.

## CONFLICT OF INTEREST

The authors have declared no conflicts of interest for this article.

## PATIENT CONSENT STATEMENT

All participants gave written informed consent.

## Supporting information


**Figure S1** Local natural frequency comparisons. Violin plots showing the distribution of the natural frequency data. (a) Distribution of the natural frequencies calculated by selecting the four channels under the stimulator as per Sarasso et al. [40]. The median is represented in the bold dashed line of each plot. The median frequency resulted from the left premotor area is 26.95 Hz, from the right premotor 29.35 Hz, from the left motor 14.20 Hz and from the right motor 14.16 Hz. Dotted lines represent the quartiles distribution of the natural frequencies for each region. (b) Distribution of the natural frequencies calculated on the one channel under the stimulator following as per Ferrarelli et al. (2012). The median is represented by the bold dashed line of each plot. The median frequency from the left premotor area is 27.20 Hz, from the right premotor 29.95 Hz, from the left motor 14.65 Hz and from the right motor 14.90 Hz. Dotted lines represent the quartiles distribution of the natural frequencies for each location.

**Figure S2** Correlation component analysis of premotor and motor TEPs. (a) Topographies and component timecourses of the principal components found applying correlated component analysis (CorrCA) in premotor TEPs. (b) Topographies and component timecourses of the principal components found applying CorrCA in motor TEPs. On the right of each panel is shown the timecourse of the most reproducible components across participants (individual participant traces in blue, average across participants in black) obtained from applying correlated component analysis (CorrCA) in the time window between 20 and 200 ms after the TMS pulse. The x axis represents the time in milliseconds, the y axis shows the voltage in microvolts. On the left of each panel is shown the topographical distribution of the forward model, which represents the sensitivity of each electrode to the component. Note that the sign (positive/negative) of the components and forward models is arbitrary. Components are sorted by the inter‐participant correlation (ISC) which measures the degree of reproducibility of the component across participants. The statistical significance of the component is assessed using surrogate statistics. For this CorrCA analysis, the channel locations of the right hemisphere stimulation were flipped right to left (see Methods).

**Figure S3** Local mean field power (LMFP) comparison between CAs. LMFP averaged across participants for each stimulated area between 20 and 200 ms from the stimulus. Kruskal–Wallis test revealed no statistically significant effect related to the stimulated area across participants (*F* = 1.08, *p* = .78). Bold dots represent the median, plain dots represent upper (above) and lower (bottom) interquartile range.

**Figure S4** Correlation analysis between TMS intensities (% MSO) and the natural frequencies. The natural frequencies for each subject are represented by a single dot along the *x* axis. The TMS intensity applied for each subject is represented in *y* axis. The black line shows the linear fitting of the distribution. (a) Correlation between stimulation intensities and natural frequencies in all the cortical areas stimulated across all the participants. (b) Correlation between stimulation intensities and natural frequencies of all the subjects derived from stimulations delivered to the left hemisphere. (c) Correlation between stimulation intensities and natural frequencies of all the subjects derived from stimulations delivered to the right hemisphere.

**Table S1** Channel selection and stimulation intensity. Sessions included in the final analysis after data preprocessing. The channels selected for the analysis are indicated, including the one closest to the stimulation site (i.e., single channel selection), and the four channels chosen for each region of interest (ROI). MSO, maximal stimulator output; TMS, transcranial magnetic stimulation.

**Table S2** Global and local natural frequency comparisons. Wilcoxon matched pairs signed rank test results obtained after comparing ipsilateral and contralateral homologous natural frequency values. These values were calculated across all participants at the global and at the local level. *p* values <.05 were deemed as significant. Stars highlight significance.

**Table S3** Comparison between natural frequencies from subjects with individual MRI available and natural frequencies from subjects with MRI template.

## Data Availability

The data that support the findings of this study are available from the corresponding author upon reasonable request.
