# Supplementary figures and images for: Physiological symmetry of transcranial magnetic stimulation‐evoked EEG spectral features
Source: Hum Brain Mapp. 2022 Jul 21;43(18):5465–77. doi: 10.1002/hbm.26022 (PMC9704783; doi:10.1002/hbm.26022)

# A

## 4 channels - ROI

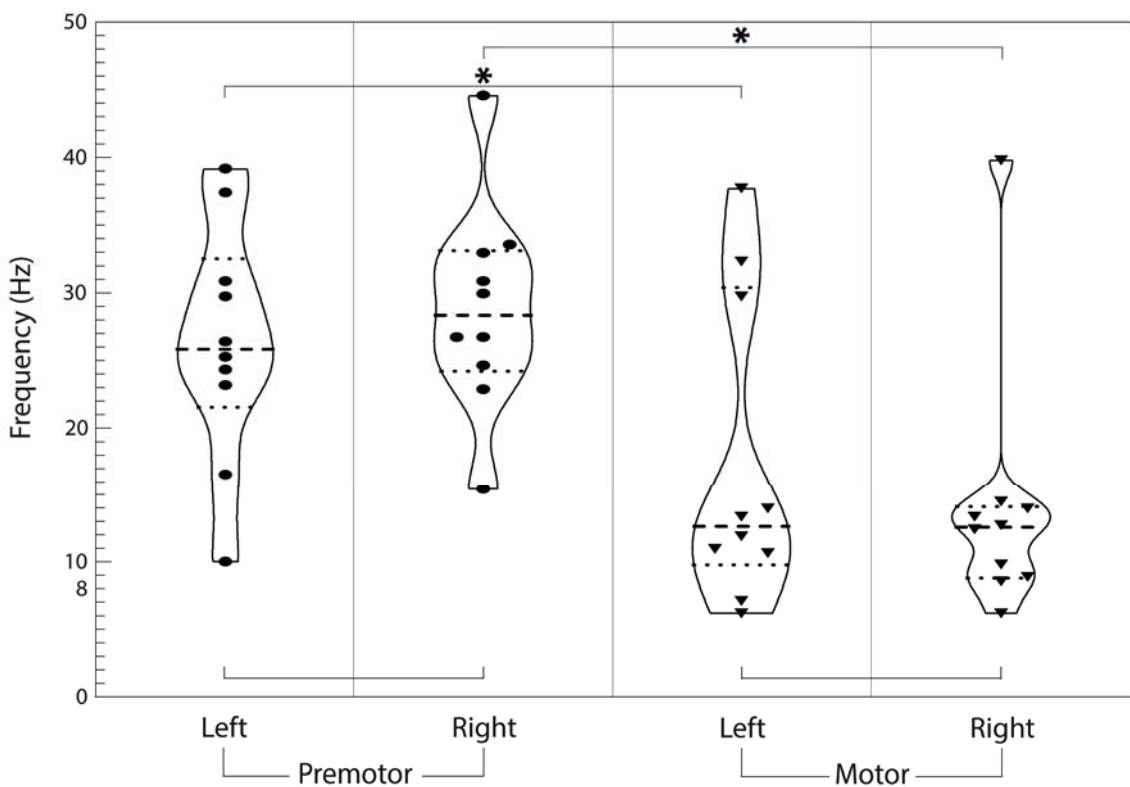

# B

## 1 channel - the closest to TMS

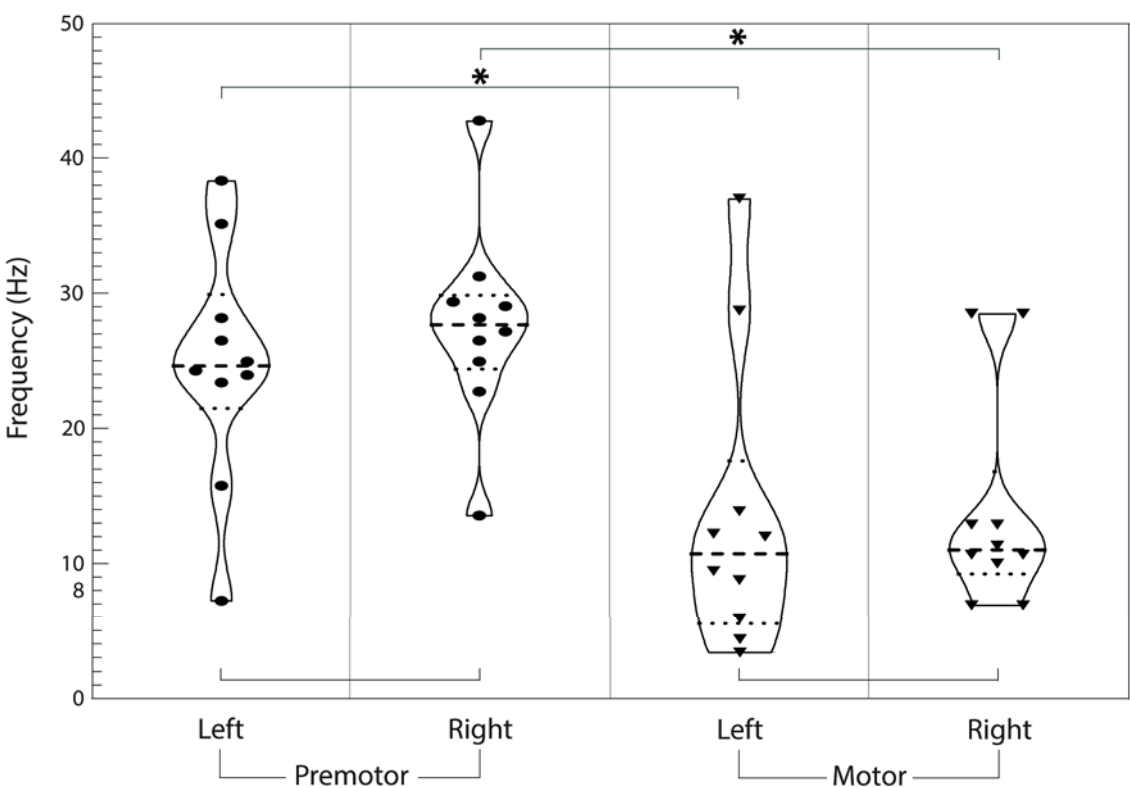

Supplement: Supplementary file 1 — Figure S1 Local natural frequency comparisons. Violin plots showing the distribution of the natural frequency data. (a) Distribution of the natural frequencies calculated by selecting the four channels under the stimulator as per Sarasso et al. [40]. The median is represented in the bold dashed line of each plot. The median frequency resulted from the left premotor area is 26.95 Hz, from the right premotor 29.35 Hz, from the left motor 14.20 Hz and from the right motor 14.16 Hz. Dotted lines represent the quartiles distribution of the natural frequencies for each region. (b) Distribution of the natural frequencies calculated on the one channel under the stimulator following as per Ferrarelli et al. (2012). The median is represented by the bold dashed line of each plot. The median frequency from the left premotor area is 27.20 Hz, from the right premotor 29.95 Hz, from the left motor 14.65 Hz and from the right motor 14.90 Hz. Dotted lines represent the quartiles distribution of the natural frequencies for each location. [file HBM-43-5465-s001.pdf]

# LMFP

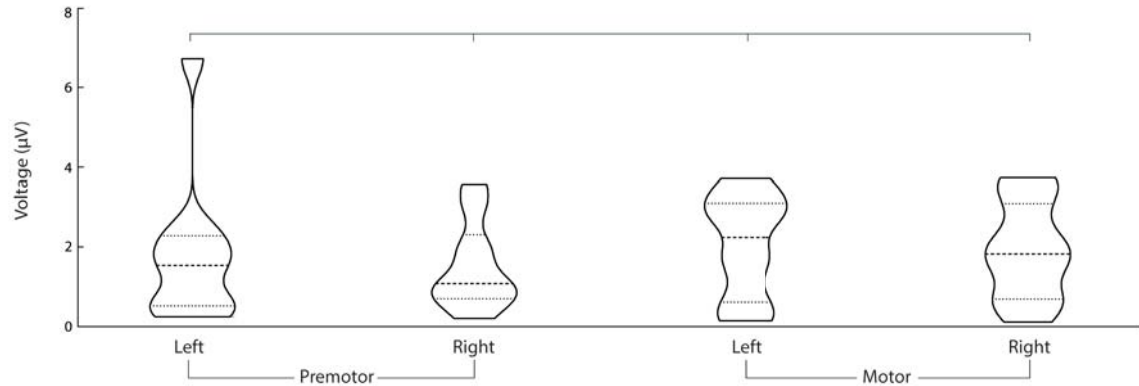

Supplement: Supplementary file 3 — Figure S3 Local mean field power (LMFP) comparison between CAs. LMFP averaged across participants for each stimulated area between 20 and 200 ms from the stimulus. Kruskal–Wallis test revealed no statistically significant effect related to the stimulated area across participants (F = 1.08, p = .78). Bold dots represent the median, plain dots represent upper (above) and lower (bottom) interquartile range. [file HBM-43-5465-s008.pdf]

**A**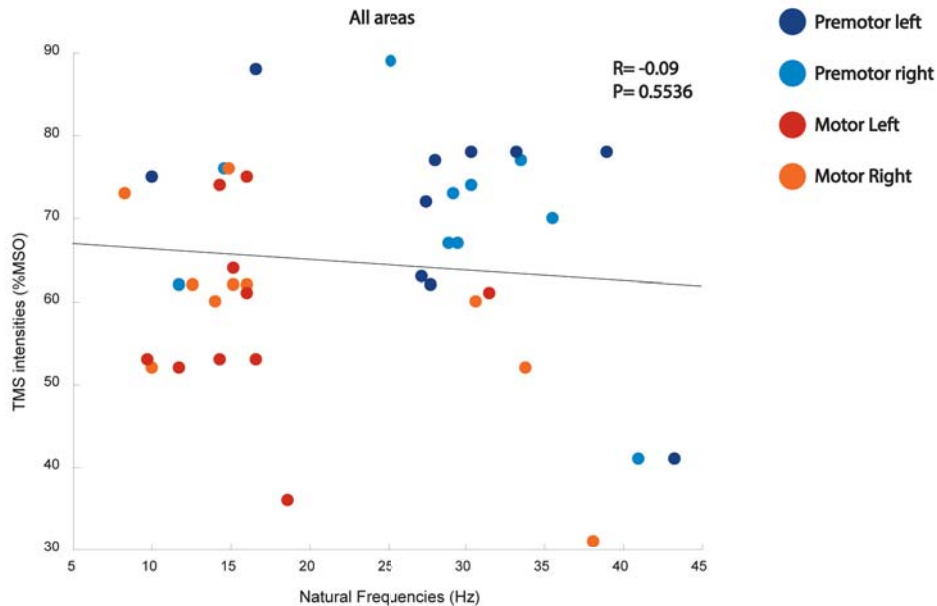**B**

**Left premotor and left motor areas**

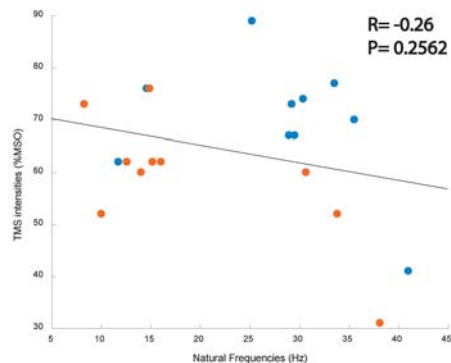**C**

**Right premotor and right motor areas**

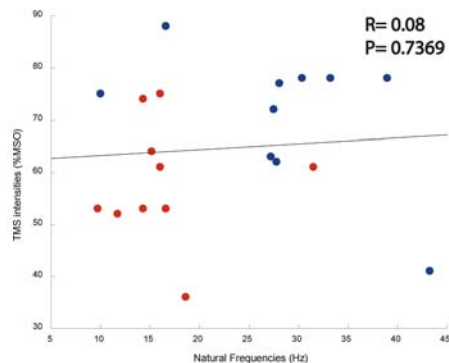

Supplement: Supplementary file 4 — Figure S4 Correlation analysis between TMS intensities (% MSO) and the natural frequencies. The natural frequencies for each subject are represented by a single dot along the x axis. The TMS intensity applied for each subject is represented in y axis. The black line shows the linear fitting of the distribution. (a) Correlation between stimulation intensities and natural frequencies in all the cortical areas stimulated across all the participants. (b) Correlation between stimulation intensities and natural frequencies of all the subjects derived from stimulations delivered to the left hemisphere. (c) Correlation between stimulation intensities and natural frequencies of all the subjects derived from stimulations delivered to the right hemisphere. [file HBM-43-5465-s002.pdf]
