# Supplementary material for: Physiological symmetry of transcranial magnetic stimulation‐evoked EEG spectral features
Source: Hum Brain Mapp. 2022 Jul 21;43(18):5465–77. doi: 10.1002/hbm.26022 (PMC9704783; doi:10.1002/hbm.26022)
Supplement: Supplementary file 2 — Figure S2 Correlation component analysis of premotor and motor TEPs. (a) Topographies and component timecourses of the principal components found applying correlated component analysis (CorrCA) in premotor TEPs. (b) Topographies and component timecourses of the principal components found applying CorrCA in motor TEPs. On the right of each panel is shown the timecourse of the most reproducible components across participants (individual participant traces in blue, average across participants in black) obtained from applying correlated component analysis (CorrCA) in the time window between 20 and 200 ms after the TMS pulse. The x axis represents the time in milliseconds, the y axis shows the voltage in microvolts. On the left of each panel is shown the topographical distribution of the forward model, which represents the sensitivity of each electrode to the component. Note that the sign (positive/negative) of the components and forward models is arbitrary. Components are sorted by the inter‐participant correlation (ISC) which measures the degree of reproducibility of the component across participants. The statistical significance of the component is assessed using surrogate statistics. For this CorrCA analysis, the channel locations of the right hemisphere stimulation were flipped right to left (see Methods). [file HBM-43-5465-s004.pdf]

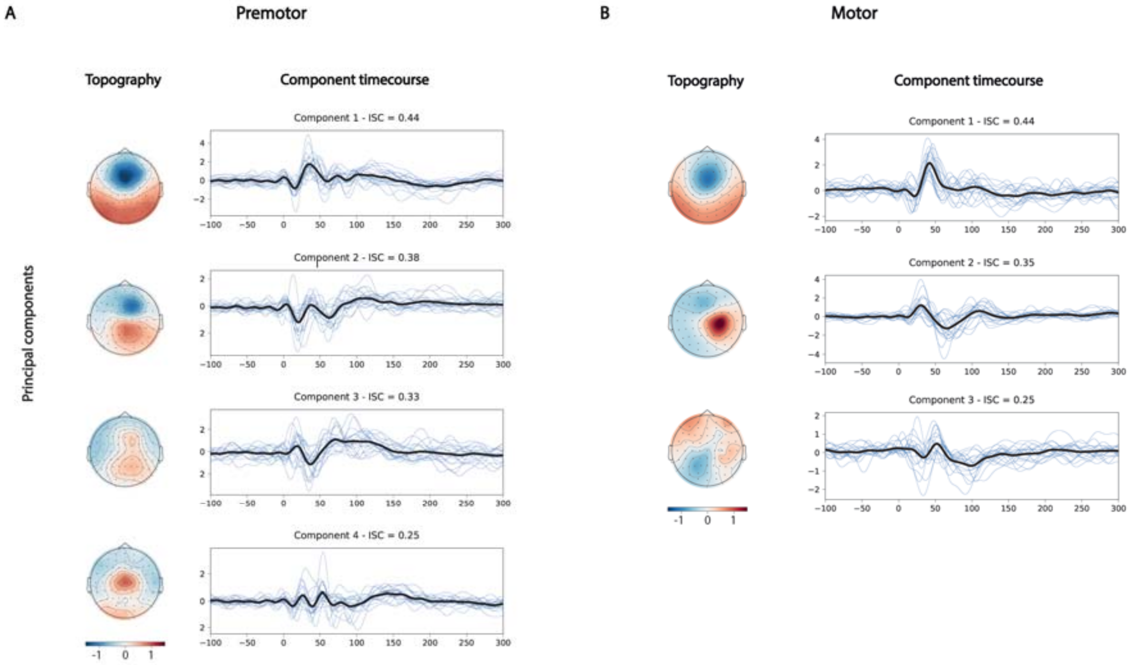
A
Premotor
B
Motor
Topography
Component timecourse
Principal components
Component 1 - ISC = 0.44
Component 2 - ISC = 0.38
Component 3 - ISC = 0.33
Component 4 - ISC = 0.25
Topography
Component timecourse
Component 1 - ISC = 0.44
Component 2 - ISC = 0.35
Component 3 - ISC = 0.25
-1 0 1
-1 0 1
