# Supplementary material for: Physiological symmetry of transcranial magnetic stimulation‐evoked EEG spectral features
Source: Hum Brain Mapp. 2022 Jul 21;43(18):5465–77. doi: 10.1002/hbm.26022 (PMC9704783; doi:10.1002/hbm.26022)
Supplement: Supplementary file 5 — Table S1 Channel selection and stimulation intensity. Sessions included in the final analysis after data preprocessing. The channels selected for the analysis are indicated, including the one closest to the stimulation site (i.e., single channel selection), and the four channels chosen for each region of interest (ROI). MSO, maximal stimulator output; TMS, transcranial magnetic stimulation. [file HBM-43-5465-s005.docx]

**Table S1. Channel selection and stimulation intensity.** Sessions included in the final analysis after data pre-processing. The channels selected for the analysis are indicated, including the one closest to the stimulation site (i.e., single channel selection), and the four channels chosen for each region of interest (ROI). TMS=Transcranial magnetic stimulation; MSO=Maximal stimulator output.

| Participants | Single channel selection / four channels selection / intensity used (MSO%) | | | |
| --- | --- | --- | --- | --- |
|  | **Left premotor** | **Right premotor** | **Left motor** | **Right motor** |
| S1 | F1/  F1-Fz-Fc1-Fcz/  89% | Fc2/  F2-Fz-Fc2-Fcz/  88% | Cp3/  C5-C3-Cp5-Cp3/ 76% | C4/  C4-C6-Cp4-Cp6/ 74% |
| S2 | Fz/  F1-Fz-Fc1-Fcz/  76% | Fc2/  F2-Fz-Fc2-Fcz/  77% | Cp3/  C5-C3-Cp5-Cp3/ 73% | Cp4/  C4-C6-Cp4-Cp6/ 75% |
| S3 | Fcz/  F1-Fz-Fc1-Fcz/ 73% | Fc2/ F2-Fz-Fc2-Fcz/ 62% | C3/  C5-C3-Cp5-Cp3/ 52% | C4/  C4-C6-Cp4-Cp6/ 53% |
| S4 | Fc1/  F1-Fz-Fc1-Fcz/ 74% | F2/ F2-Fz-Fc2-Fcz/ 72% | Cp3/  C5-C3-Cp5-Cp3/ 60% | C4/  C4-C6-Cp4-Cp/ 61% |
| S5 | Fc1/  F1-Fz-Fc1-Fcz/ 77% | Fc2/  F2-Fz-Fc2-Fcz/ 78% | Cp3/  C5-C3-Cp5-Cp3/ 62% | Cp4/  C4-C6-Cp4-Cp6/ 64% |
| S6 | F1/  F1-Fz-Fc1-Fcz/ 67% | Fc2/  F2-Fz-Fc2-Fcz/ 78% | Cp3/  C5-C3-Cp5-Cp3/ 62% | C4/  C4-C6-Cp4-Cp6/ 53% |
| S7 | F1/  F1-Fz-Fc1-Fcz/ 62% | F2/ F2-Fz-Fc2-Fcz/ 63% | C3/  C5-C3-Cp5-Cp3/ 52% | C4/  C4-C6-Cp4-Cp6/ 53% |
| S8 | F1/  F1-Fz-Fc1-Fcz/  70% | F2/ F2-Fz-Fc2-Fcz/ 75% | Cp5/  C5-C3-Cp5-Cp3/ 60% | C4/  C4-C6-Cp4-Cp/ 61% |
| S9 | F1/  F1-Fz-Fc1-Fcz/ 67% | Fc2/  F2-Fz-Fc2-Fcz/ 78% | Cp3/  C5-C3-Cp5-Cp3/ 62% | C4/  C4-C6-Cp4-Cp6/ 53% |
| S10 | Fc1/  F1-Fz-Fc1-Fcz/ 41% | Fc2/  F2-Fz-Fc2-Fcz/ 41% | Cp3/  C5-C3-Cp5-Cp3/ 31% | Cp6/  C4-C6-Cp4-Cp6/ 36% |
| Median stimulation intensity* | 71.50% | 76.00% | 61.00% | 57.00 |

*The stimulation intensity is reported as maximal stimulator output (MSO) percentage**.**
