# Supplementary material for: Physiological symmetry of transcranial magnetic stimulation‐evoked EEG spectral features
Source: Hum Brain Mapp. 2022 Jul 21;43(18):5465–77. doi: 10.1002/hbm.26022 (PMC9704783; doi:10.1002/hbm.26022)
Supplement: Supplementary file 6 — Table S2 Global and local natural frequency comparisons. Wilcoxon matched pairs signed rank test results obtained after comparing ipsilateral and contralateral homologous natural frequency values. These values were calculated across all participants at the global and at the local level. p values <.05 were deemed as significant. Stars highlight significance. [file HBM-43-5465-s007.docx]

**Table S2. Global and local natural frequency comparisons.** Wilcoxon matched pairs signed rank test results obtained after comparing ipsilateral and contralateral homologous natural frequency values. These values were calculated across all participants at the global and at the local level. P-values <0.05 were deemed as significant. Stars highlight significance.

| **Natural frequencies comparison** | | | | |
| --- | --- | --- | --- | --- |
| **Channel Selection** | **Contralateral homologous comparisons** | | **Ipsilateral comparisons** | |
|  | Left vs. right premotor | Left vs. right motor | Left premotor vs. motor | Right premotor vs. motor |
| All channels | *P=*0.6426, (*W=*10 *P=*0.6426 | *W*=-6  *P=*0.7871, (*W* =-6.00) | *W*=-45  *P=*0.0195* | *W*=-39  *P=*0.0488* |
| Four channels | *W*=11  *P=*0.5703, (*W=11.00* ) | *W*=-3  *P=*0.9102, (*W=-3.00*) | *W*=41  *P=*0.0371* | *W*=-55  *P=*0.002* |
| One channel | *W*=15  *P=*0.4258, (*W=15.00* ) | *W*=10  *P=*0.6309 | *W*=-47  *P=*0.0137* | *W*=-51  *P=*0.0059* |
