# Supplementary material for: Physiological symmetry of transcranial magnetic stimulation‐evoked EEG spectral features
Source: Hum Brain Mapp. 2022 Jul 21;43(18):5465–77. doi: 10.1002/hbm.26022 (PMC9704783; doi:10.1002/hbm.26022)
Supplement: Supplementary file 7 — Table S3 Comparison between natural frequencies from subjects with individual MRI available and natural frequencies from subjects with MRI template. [file HBM-43-5465-s003.docx]

**Table S3.** Comparison between natural frequencies from subjects with individual MRI available and natural frequencies from subjects with MRI template

| **Area** | **Premotor Left** | **Premotor Right** | **Motor Left** | **Motor Right** |
| --- | --- | --- | --- | --- |
| P (value) | 0.43 | 0.12 | 0.06 | 0.06 |
| W (Wilcoxon test) | 7 | 13 | 15 | 15 |
