# Supplementary material for: Physiological symmetry of transcranial magnetic stimulation‐evoked EEG spectral features
Source: Hum Brain Mapp. 2022 Jul 21;43(18):5465–77. doi: 10.1002/hbm.26022 (PMC9704783; doi:10.1002/hbm.26022)
Supplement: Supplementary file 8 [file HBM-43-5465-s006.docx]

**Supplementary Material of the work “Physiological symmetry of**

**TMS-evoked EEG potentials”.**

Sasha D’Ambrosio*^1,2^, Diego Jiménez-Jiménez*^1,2^, Katri Silvennoinen^1,3^, Sara Zagaglia^1^, Marco Perulli^1,4^, Josephine Poole^1^, Renzo Comolatti^5^, Matteo Fecchio^6^, Sanjay M. Sisodiya^1^, Simona Balestrini^1,7^

*These authors contributed equally to this work

1 Department of Clinical and Experimental Epilepsy, UCL Queen Square Institute of Neurology, London, UK

2 The Chalfont Centre for Epilepsy, Chalfont St. Peter, UK

3 Neuro Center, Kuopio University Hospital, Kuopio, Finland

4 Department of Neuroscience, Catholic University of the Sacred Heart, Rome, Italy

5 Dipartimento di Scienze Biomediche e Cliniche "L. Sacco", Università degli Studi di Milano, Milan, Italy

6 Center for Neurotechnology and Neurorecovery, Department of Neurology, Massachusetts General Hospital, Boston, Massachusetts, USA

7 Neuroscience Department, Meyer Children's Hospital-University of Florence, Florence, Italy

**Methods**

**S1. The Chalfont transcranial magnetic stimulation (TMS)-EEG protocol**

In order to deliver TMS in a systematic manner we have developed the following protocol which was used for all the participants included in the study in all the different cortical areas (CAs) stimulated. A graphic representation of the protocol is presented in Figure 1. This protocol includes thirteen steps, which are labelled in roman numerals. The steps to follow vary according to the different CA stimulated, as each CA requires different parameters in order to obtain meaningful and reproducible responses.

**I.** **Target the region of interest in 3-D.** Upload the whole head T1 – weighted structural Magnetic Resonance Image (MRI) of the participant (if available) or the template, on the navigation software. Choose the cardinal targets, pre-auricular points, the nasion and the tip of the nose. Once done, select your region of interest for the stimulation. Specifically, in this work: BA6 for premotor stimulations, BA4 for motor stimulations

**II.** **Fit the cap and attach EMG electrodes.** Measure the participant’s head according to the international guidelines to select the appropriate size for the EEG cap [1]. Choose a cap that fits the participant’s head correctly. Ensure that all electrodes are adjacent to the scalp. Attach the EMG electrodes. Place two disposable electrodes on the first dorsal interosseus muscle (FDI) contralateral to the hemisphere under study, using bipolar surface disposable solid gel electrodes (size: 15x20 mm).

**III.** **Lower the impedances.** Using a disposable curved syringe, adjust the blunt tip of the syringe and fill it with an electroconductive gel. Put the curved tip inside the hole of the electrode, and then press the plunger flange until there is some gel on the skin. Mildly scrub the scalp with the syringe tip using circular movements. Make sure that the gel is not spilling out over the electrode to avoid any bridging phenomenon. Measure the impedance of each channel. When all channel impedances are below 5kΩ, move to the next [2].

**IV.** **Brain navigation co-registration.** Locate the head tracker without interfering with the coil or the electrodes. Using the digitizing pen, co-register the cardinal points selected earlier on the participant's head (either on MRI or template). Once the coordinates are accurate, proceed to create a 3-D MRI model and validate the co-registration (we suggest plugging the noise-cancelling earphones into the participants ears at this stage; see Methods: “TMS-click sound masking”).

**V.**  **Record resting EEG before TMS**. Record two separate resting EEGs of 5–10 minutes, one asking the participant to stare at a fixed point with their eyes open, the other asking the participant to close their eyes and be relaxed. EEG should be recorded according to the international standard criteria [1].

**VI.** **Measure the resting motor threshold (RMT).** Ask the participant to remain relaxed. By moving the coil within the region of interest (ROI), search for the ‘hotspot’ able to elicit a twitch only on the first dorsal interosseous muscle (FDI). Using different TMS intensities, find the one that evoke motor-evoked potentials (MEPs) of 50µV over FDI in at least five of ten pulses delivered at a minimum frequency of 0.2Hz.

**VII.** **Find an artefact-free target by online TEP visualisation.** Load the target area on the neuronavigation software. Ask the participant to remain relaxed. Depending on the stimulated area, select an appropriate stimulation intensity accordingly: a) for premotor stimulation start at intensities close to the RMT; b) for motor stimulation start at ≤90% of the RMT. By the end of this step the operator should have found a stimulation target that does not elicit muscle activity on the EEG nor in the body of the participant. Moreover, there should be no electrical artifact lasting more than 10ms, excluding the TMS pulse. Take a note of the best target coordinates.

**VIII. Keep intensity** ≤**90% RMT.** For motor cortex stimulations only: perform step IX keeping the stimulator intensity below ≤90% of the RMT, in order to avoid sensory feedback contamination [3].

**IX.** **Determine the stimulation intensity by averaging 20 trials.** Select the coordinates resulting from VII as stimulation target within the neuronavigator. Ask the participant to remain relaxed staring at a fixed position. For motor stimulation deliver 20 pulses keeping the stimulator intensity below ≤90% of the RMT. For premotor stimulation deliver 20 pulses, then measure the amplitude, in average reference, of the response in the channel closest to the stimulation site. If the first component after 10ms from the TMS pulse measures ≥10µV peak-to-peak in average reference, then take note of the intensity used. Otherwise, tune the intensity accordingly and repeat the 20 trials delivery. If no electrical artifacts (apart from the one from the TMS pulse) are present and no MEP is present during motor stimulation, move to step X. Otherwise go back to step VII.

**X.** **Check the impedances.** Check the impedances for all the electrodes and lower them according to III.

**XI.** **Start recording at least 150 trials.** Ask the participant to remain in a comfortable position. Here we suggest to start the noise masking (see main Methods section “*TMS-click sound masking*”). Locate the coil aiming at the target noted in VII. Ensure not to push the electrodes with the coil. Use the intensity as determined in step IX. Start recording EEG. Deliver at least 150 pulses. During motor stimulation, monitor the presence of MEP: if no MEPs are elicited during motor stimulations, then move to step XII. Otherwise, stop the session and re-start from IX lowering the stimulation intensity.

**XII.** **Record resting EEG after TMS.** Record two separate resting EEGs of 5-10 minutes, one asking the participant to stare at a fixed point with their eyes open, the other asking the participant to close their eyes and be relaxed. The EEG should be recorded according to the international standard criteria [1].

**XIII.** **End of the experimental session.** Once the data has been collected, gently remove the electrodes, earphones, EEG cap and save the data.

**S2. TMS-click sound masking.**

During each experimental session, we delivered a noise-masking sound of at least 37dB characterised by a white noise and a simultaneous sound that has the same spectral profile as the TMS coil click. To this aim, we used a noise-masking tool that is fine-tuned for each participant in regards of their individual perception [4]. Before starting each session, the volume of the masking noise was gradually increased until the participant could not hear the TMS coil click. If participants reported hearing any TMS clicks during a session, the session was re-started with an increased volume of the masking noise. The volume was always kept at a comfortable level for the participant [5–7], and never above 85dB, to prevent adverse effects [8].

**S3. EMG recording and RMT measurement**

Electromyographic (EMG) recordings were obtained from the FDI muscle using bipolar surface electrodes (15x20mm disposable solid gel electrodes, Unimed Electrode Supplies Limited, United Kingdom) placed approximately 3cm apart in a belly tendon montage. A common ground electrode was placed over the fourth metacarpal. EMG signals were amplified (0.1mV), band-pass filtered (high pass 5Hz, low pass 2kHz, notch 50Hz) and digitised at 5kHz with a Signal interface system (Cambridge Electronic Design Co. Ltd., United Kingdom).

**S4. Natural frequencies comparison by means of global and local responses.**

To quantify the responses in the time frequency domain from each TMS-EEG session in our cohort, we measured the event-related spectral perturbation (ERSP) changes in natural frequencies globally and locally. Specifically, natural frequencies were calculated globally as described in the main Methods section [6]. Moreover, we calculated the natural frequency locally, by measuring natural frequencies across the four channels under the stimulator (see ROI in Table S1) [9] and at the channel closest to the stimulation site [10] (see the single channel selection in Table S1). Comparison of natural frequencies was performed by using Wilcoxon test. P-values <0.05 were deemed as significant.

**S5. CorrCA on individual CAs across participants.**

Correlated component analysis is a technique introduced in 2012 [11] to identify reliable components in multidimensional data that are reproducible across repeated measurements. In data structured as NxDxT, where T are time samples, D the sensors, and N are repeated voltage measures per sensors, the method extracts directions in the D-dimensional data by maximizing the correlation between N repeated measurements (e.g. participants, trials, etc), where correlation is calculated across T samples [12]. More specifically, instead of finding dimensions that maximize the variance present in the data as in Principal Component Analysis (PCA), this method finds dimensions where the ratio between the correlation among repetitions and the correlation within repetitions is maximal (see [13] for the further details), thus identifying components which are reproducible across repetitions. This normalized ratio associated to each identified component is called inter-participant correlation (ISC), ranges from 0 to 1, and measures the degree of reproducibility of the component across the N repetitions. The components are ranked by ISC and their statistical significance is tested non-parametrically using surrogates obtained by a random circular shuffle in the data that preserves individual within-participant correlation but not the correlation across repetitions [13].

**Results:**

**S6. Natural frequencies comparison by means of local responses.**

At the local level, we found statistically significant differences among ipsilateral comparison of natural frequencies, whereas we did not find significant differences between homologous contralateral comparisons (Figure S1, Table S2). These results are in line with the comparison performed on the global responses, reported in the main results section.

**S7. Correlation coefficient**

1. **Pairwise comparisons**

Multiple comparisons of Rs using Dunn's test revealed significant differences between left ipsilateral vs. premotor contralateral homologous Rs (Mean rank difference = -18.80, P=0.0019), left ipsilateral vs. motor contralateral homologous Rs (Mean rank difference = -18.20, P=0.0030), right ipsilateral vs. premotor contralateral homologous Rs (Mean rank difference = -18.20, P=0.0030) and right ipsilateral vs. motor contralateral homologous Rs (Mean rank difference =-17.60, P=0.0046). Premotor contralateral homologous vs. motor contralateral homologous Rs comparisons were not statistically significant (Mean rank difference =0.600, P>0.999) as well as left ipsilateral vs. right ipsilateral comparisons (Mean rank difference =-0.600, P>0.999). P-values <0.05 were deemed as significant.

1. **Association between TMS intensity and natural frequency**

We computed the correlation coefficient between stimulation intensities and TEP natural frequencies. We then applied the same analysis after dividing our cohort between left and right hemispheric stimulation, therefore comparing left premotor vs motor TEPs, and right premotor vs motor TEPs separately. Lastly, we conducted a linear regression analysis of the natural frequency distribution (y= α+βx, where y is intensity and x the natural frequency) to further analyse the association between the intensity and natural frequencies. P-values <0.05 were deemed as significant. When we calculated the correlation coefficient among all TEPs, we found an R=-0.09 (P=0.5536), and beta coefficient of the linear regression (β)=–0.12 (P=0.5536, Figure S4, A). The correlation coefficient for left areas was R=-0.26 (P=0.2562), the beta coefficient was β=–0.33 (P=0.2562, Figure S4, B). The correlation coefficient for right areas was R=-0.08 (P=0.7369), the beta coefficient was β=0.11 (P=0.7369, Figure S4, C). We did not find any significant correlation between intensities and natural frequencies.

**S8. Bilateral CorrCA across participants**

To assess the reproducibility of TEP components bilaterally across participants, CorrCA was applied to premotor and motor TEPs merging the signals from the two hemispheres. To this aim we combined TEPs by flipping the channel location of the right premotor and right motor recordings to the left. In this case, we computed CorrCA between 20 and 200 ms from the TMS pulse on “20 left premotor” (10 left premotor + 10 right premotor flipped to the left) and “20 left motor” (10 left motor + 10 right motor flipped to the left). Interestingly, premotor TEPs were characterised by four statistically significant components. Specifically, the most reproducible component (inter-participant correlation (ISC)=0.44) showed three peaks within the first 100ms and was located in the premotor area in the midline. The second component (ISC=0.38) showed two peaks within the first 100ms and was located between the premotor and the motor area. The third component (ISC=0.33) showed two peaks within the first 100ms and was located between the premotor and the motor area. The fourth component (ISC=0.25) showed three peaks within the first 100ms and was located between the premotor and the motor area in the midline (Figure S2, panel A).

Motor TEPs were characterized by three statistically significant components. The most reproducible (ISC=0.44) component showed one peak within the first 100ms and was located in the midline. The second component (ISC=0.35) presented one peak within the first 100ms and it was located in the motor area contralateral to the stimulated target. Finally, the third component (ISC=0.25) showed one peak within the first 100ms and was located over the ipsilateral parietal region (Figure S2, panel B).

**S9. Local mean field power**

The local mean field power (LMFP) is a measure of brain activation effectively evoked by TMS at the local level [14]. LMFP is computed as the square root of the summed squared voltage values of TEPs across a given ROI [15]. To estimate significant LMFP values compared with baseline, we followed previously described methodology [14]. We selected a window between -500 to -100ms from the stimulus as baseline in order to remain sufficiently far from trial split limits and post-stimulus. Thus, we avoided possible edge artifacts and post-stimulus contamination of the baseline, according to previously reported methods [3]. We computed bootstrap statistics in every segment that was obtained after pre-processing each session, in each ROI. Specifically, we shuffled the pre-stimulus activity at the single trial level, obtaining 1000 (α < 0.01) surrogate time-series. Thus, we calculated the maximal distribution as the maximum values from each random surrogate across all latencies. Subsequently, for each TEP, we summed the significant values (significance level = p<0.01) of the mean of the trials in the time window between 20 and 200ms after the stimulus [3]. Finally, we compared LMFPs across TEPs by applying the Kruskal-Wallis test. P-values <0.05 were deemed as significant (Figure S3).

**S10. Relative distances**

We measured the relative distances between each stimulation site in order to control for the location of the stimulations as potential confounders of both gradient and symmetry of TEPs. Specifically, we recorded the 3-dimensional stimulation coordinates during each session. We then calculated the 3-dimensional Euclidean distance (the sum of the absolute values of the difference of each dimension). Therefore, for each participant, we measured the distance between both the two ipsilateral and the two contralateral homologous stimulation sites (Figure 5, panel A). We performed the Wilcoxon matched pairs signed rank test for comparing each relative distance across all participants. P-values <0.05 were deemed as significant.

**S11. Interhemispheric signal propagation (ISP) and inter hemispheric balance (IHB).**

We found ISP <1 (median 0.41, IQR 0.24) for motor left stimulations, as well as <1 for motor right stimulations (median 0.38, IQR 0.286). We did not find any significant difference after comparing ISP between the two hemispheres (W=-1.00, P=0.999). IHB also showed a consistent interhemispheric balance between left motor and right motor cortices (median 0.98, IQR 0.55). Due to the anatomical targets of our protocol, we were not able to calculate ISP and IHB for premotor TEPs. In fact, the proximity to the midline of our premotor targets resulted in an overlap of the channels required for these calculations. Of note, we found similar TEP properties in the unilateral analysis as in the bilateral that we present in the main results section.

**References**

[1] Nuwer MR, Comi G, Emerson R, Fuglsang-Frederiksen A, Guerit M, Hinrichs H, et al. IFCN standards for digital recording of clinical EEG. Electroencephalogr Clin Neurophysiol 1998. https://doi.org/10.1016/S0013-4694(97)00106-5.

[2] Ilmoniemi RJ, Kičić D. Methodology for combined TMS and EEG. Brain Topogr 2010. https://doi.org/10.1007/s10548-009-0123-4.

[3] Fecchio M, Pigorini A, Comanducci A, Sarasso S, Casarotto S, Premoli I, et al. The spectral features of EEG responses to transcranial magnetic stimulation of the primary motor cortex depend on the amplitude of the motor evoked potentials. PLoS One 2017. https://doi.org/10.1371/journal.pone.0184910.

[4] Russo S, Sarasso S, Puglisi GE, Dal Palù D, Pigorini A, Casarotto S, et al. TAAC - TMS Adaptable Auditory Control: a universal tool to mask TMS click. J Neurosci Methods 2022:109491. https://doi.org/10.1016/j.jneumeth.2022.109491.

[5] Rosanova M, Fecchio M, Casarotto S, Sarasso S, Casali AG, Pigorini A, et al. Sleep-like cortical OFF-periods disrupt causality and complexity in the brain of unresponsive wakefulness syndrome patients. Nat Commun 2018. https://doi.org/10.1038/s41467-018-06871-1.

[6] Rosanova M, Casali A, Bellina V, Resta F, Mariotti M, Massimini M. Natural frequencies of human corticothalamic circuits. J Neurosci 2009. https://doi.org/10.1523/JNEUROSCI.0445-09.2009.

[7] Fuggetta G, Fiaschi A, Manganotti P. Modulation of cortical oscillatory activities induced by varying single-pulse transcranial magnetic stimulation intensity over the left primary motor area: A combined EEG and TMS study. Neuroimage 2005. https://doi.org/10.1016/j.neuroimage.2005.05.013.

[8] Berglund B, Lindvall T, Schwela DH. New Who Guidelines for Community Noise. Noise Vib Worldw 2000. https://doi.org/10.1260/0957456001497535.

[9] Sarasso S, D’Ambrosio S, Fecchio M, Casarotto S, Viganò A, Landi C, et al. Local sleep-like cortical reactivity in the awake brain after focal injury. Brain 2020. https://doi.org/10.1093/brain/awaa338.

[10] Ferrarelli F, Sarasso S, Guller Y, Riedner BA, Peterson MJ, Bellesi M, et al. Reduced natural oscillatory frequency of frontal thalamocortical circuits in schizophrenia. Arch Gen Psychiatry 2012. https://doi.org/10.1001/archgenpsychiatry.2012.147.

[11] Dmochowski J, Sajda P, Dias J, Parra L. Correlated Components of Ongoing EEG Point to Emotionally Laden Attention – A Possible Marker of Engagement? Front Hum Neurosci 2012;6:112. https://doi.org/10.3389/fnhum.2012.00112.

[12] Parra LC, Haufe S, Dmochowski JP. Correlated Components Analysis - Extracting Reliable Dimensions in Multivariate Data 2018.

[13] Delorme A, Makeig S. EEGLAB: An open source toolbox for analysis of single-trial EEG dynamics including independent component analysis. J Neurosci Methods 2004. https://doi.org/10.1016/j.jneumeth.2003.10.009.

[14] Casarotto S, Canali P, Rosanova M, Pigorini A, Fecchio M, Mariotti M, et al. Assessing the effects of electroconvulsive therapy on cortical excitability by means of transcranial magnetic stimulation and electroencephalography. Brain Topogr 2013. https://doi.org/10.1007/s10548-012-0256-8.

[15] Lehmann D, Skrandies W. Reference-free identification of components of checkerboard-evoked multichannel potential fields. Electroencephalogr Clin Neurophysiol 1980. https://doi.org/10.1016/0013-4694(80)90419-8.

**Supplementary Tables**

**Table S1. Channel selection and stimulation intensity.** Sessions included in the final analysis after data pre-processing. The channels selected for the analysis are indicated, including the one closest to the stimulation site (i.e., single channel selection), and the four channels chosen for each region of interest (ROI). TMS=Transcranial magnetic stimulation; MSO=Maximal stimulator output.

| Participants | Single channel selection / four channels selection / intensity used (MSO%) | | | |
| --- | --- | --- | --- | --- |
|  | **Left premotor** | **Right premotor** | **Left motor** | **Right motor** |
| S1 | F1/  F1-Fz-Fc1-Fcz/  89% | Fc2/  F2-Fz-Fc2-Fcz/  88% | Cp3/  C5-C3-Cp5-Cp3/ 76% | C4/  C4-C6-Cp4-Cp6/ 74% |
| S2 | Fz/  F1-Fz-Fc1-Fcz/  76% | Fc2/  F2-Fz-Fc2-Fcz/  77% | Cp3/  C5-C3-Cp5-Cp3/ 73% | Cp4/  C4-C6-Cp4-Cp6/ 75% |
| S3 | Fcz/  F1-Fz-Fc1-Fcz/ 73% | Fc2/ F2-Fz-Fc2-Fcz/ 62% | C3/  C5-C3-Cp5-Cp3/ 52% | C4/  C4-C6-Cp4-Cp6/ 53% |
| S4 | Fc1/  F1-Fz-Fc1-Fcz/ 74% | F2/ F2-Fz-Fc2-Fcz/ 72% | Cp3/  C5-C3-Cp5-Cp3/ 60% | C4/  C4-C6-Cp4-Cp/ 61% |
| S5 | Fc1/  F1-Fz-Fc1-Fcz/ 77% | Fc2/  F2-Fz-Fc2-Fcz/ 78% | Cp3/  C5-C3-Cp5-Cp3/ 62% | Cp4/  C4-C6-Cp4-Cp6/ 64% |
| S6 | F1/  F1-Fz-Fc1-Fcz/ 67% | Fc2/  F2-Fz-Fc2-Fcz/ 78% | Cp3/  C5-C3-Cp5-Cp3/ 62% | C4/  C4-C6-Cp4-Cp6/ 53% |
| S7 | F1/  F1-Fz-Fc1-Fcz/ 62% | F2/ F2-Fz-Fc2-Fcz/ 63% | C3/  C5-C3-Cp5-Cp3/ 52% | C4/  C4-C6-Cp4-Cp6/ 53% |
| S8 | F1/  F1-Fz-Fc1-Fcz/  70% | F2/ F2-Fz-Fc2-Fcz/ 75% | Cp5/  C5-C3-Cp5-Cp3/ 60% | C4/  C4-C6-Cp4-Cp/ 61% |
| S9 | F1/  F1-Fz-Fc1-Fcz/ 67% | Fc2/  F2-Fz-Fc2-Fcz/ 78% | Cp3/  C5-C3-Cp5-Cp3/ 62% | C4/  C4-C6-Cp4-Cp6/ 53% |
| S10 | Fc1/  F1-Fz-Fc1-Fcz/ 41% | Fc2/  F2-Fz-Fc2-Fcz/ 41% | Cp3/  C5-C3-Cp5-Cp3/ 31% | Cp6/  C4-C6-Cp4-Cp6/ 36% |
| Median stimulation intensity* | 71.50% | 76.00% | 61.00% | 57.00 |

*The stimulation intensity is reported as maximal stimulator output (MSO) percentage**.**

**Table S2. Global and local natural frequency comparisons.** Wilcoxon matched pairs signed rank test results obtained after comparing ipsilateral and contralateral homologous natural frequency values. These values were calculated across all participants at the global and at the local level. P-values <0.05 were deemed as significant. Stars highlight significance.

| **Natural frequencies comparison** | | | | |
| --- | --- | --- | --- | --- |
| **Channel Selection** | **Contralateral homologous comparisons** | | **Ipsilateral comparisons** | |
|  | Left vs. right premotor | Left vs. right motor | Left premotor vs. motor | Right premotor vs. motor |
| All channels | *P=*0.6426, (*W=*10 *P=*0.6426 | *W*=-6  *P=*0.7871, (*W* =-6.00) | *W*=-45  *P=*0.0195* | *W*=-39  *P=*0.0488* |
| Four channels | *W*=11  *P=*0.5703, (*W=11.00* ) | *W*=-3  *P=*0.9102, (*W=-3.00*) | *W*=41  *P=*0.0371* | *W*=-55  *P=*0.002* |
| One channel | *W*=15  *P=*0.4258, (*W=15.00* ) | *W*=10  *P=*0.6309 | *W*=-47  *P=*0.0137* | *W*=-51  *P=*0.0059* |

**Table S3. Comparison between individual MRI vs template and natural frequencies.** Wilcoxon matched pairs signed rank test results obtained after comparing the distributions of natural frequencies derived from TEPs recorded in subjects with individual MRI vs. the natural frequencies derived from TEPs recorded in subjects where template MRI was used. P value was deemed to be statistically significant <0.005. In the middle of the table, we added the statistic value for the Wilcoxon Signed Rank Test (W) and the P value (P).

| **Area** | **Left Premotor** | **Right Premotor** | **Left Motor** | **Right Motor** |
| --- | --- | --- | --- | --- |
| P (value) | 0,43 | 0,12 | 0,06 | 0,06 |
| W (Wilcoxon test) | 7 | 13 | 15 | 15 |

**Supplementary Figure Legends**

**Figure S1. Local natural frequency comparisons.** Violin plots showing the distribution of the natural frequency data. **A.** Distribution of the natural frequencies calculated by selecting the four channels under the stimulator as per Sarasso et al [40]. The median is represented in the bold dashed line of each plot. The median frequency resulted from the left premotor area is 26.95Hz, from the right premotor 29.35Hz, from the left motor 14.20Hz and from the right motor 14.16Hz. Dotted lines represent the quartiles distribution of the natural frequencies for each region. **B.** Distribution of the natural frequencies calculated on the one channel under the stimulator following as per Ferrarelli et al. [10]. The median is represented by the bold dashed line of each plot. The median frequency from the left premotor area is 27.20Hz, from the right premotor 29.95Hz, from the left motor 14.65Hz and from the right motor 14.90Hz. Dotted lines represent the quartiles distribution of the natural frequencies for each location.

**Figure S2. Correlation component analysis of premotor and motor TEPs. A**. Topographies and component timecourses of the principal components found applying correlated component analysis (CorrCA) in premotor TEPs. **B.** Topographies and component timecourses of the principal components found applying CorrCA in motor TEPs. On the right of each panel is shown the timecourse of the most reproducible components across participants (individual participant traces in blue, average across participants in black) obtained from applying correlated component analysis (CorrCA) in the time window between 20 and 200ms after the TMS pulse. The x axis represents the time in milliseconds, the y axis shows the voltage in microvolts. On the left of each panel is shown the topographical distribution of the forward model, which represents the sensitivity of each electrode to the component. Note that the sign (positive/negative) of the components and forward models is arbitrary. Components are sorted by the inter-participant correlation (ISC) which measures the degree of reproducibility of the component across participants. The statistical significance of the component is assessed using surrogate statistics. For this CorrCA analysis, the channel locations of the right hemisphere stimulation were flipped right to left (see methods).

**Figure S3. Local mean field power (LMFP) comparison between CAs.** LMFP averaged across participants for each stimulated area between 20 and 200ms from the stimulus. Kruskal-Wallis test revealed no statistically significant effect related to the stimulated area across participants (F=1.08, P=0.78). Bold dots represent the median, plain dots represent upper (above) and lower (bottom) interquartile range.

**Figure S4. Correlation analysis between TMS intensities (% MSO) and the natural frequencies.** The natural frequencies for each subject are represented by a single dot along the X axis. The TMS intensity applied for each subject is represented in y axis. The black line shows the linear fitting of the distribution. **A.** Correlation between stimulation intensities and natural frequencies in all the cortical areas stimulated across all the participants. **B.** Correlation between stimulation intensities and natural frequencies of all the subjects derived from stimulations delivered to the left hemisphere. **C.** Correlation between stimulation intensities and natural frequencies of all the subjects derived from stimulations delivered to the right hemisphere.
